# A guard cell carbonic anhydrase binds and regulates SLAC1 separate from its catalytic activity

**DOI:** 10.1038/s41467-026-70596-9

**Published:** 2026-03-13

**Authors:** Lingfeng Xia, Jonas Chaves Alvim, Thanh-Hao Nguyen, Cecile Lefoulon, Fernanda A. L. Silva-Alvim, Zhiyi Yu, Martina Klejchova, Sahar Farami, Sakharam Waghmare, Rucha Karnik, Michael R. Blatt

**Affiliations:** 1 Laboratory of Plant Physiology and Biophysics, Bower Building, University of Glasgow, Glasgow, G12 8QQ UK; 2 The School of Molecular Biosciences, Bower Building, University of Glasgow, Glasgow, G12 8QQ UK

**Keywords:** Stomata, Plant signalling, Plant cell biology

## Abstract

Stomata of plant leaves open to enable CO_2_ entry for photosynthesis and close when CO_2_ in the leaf is elevated. CO_2_ is thought to promote stomatal closure in part by activating the SLAC1 anion channel at the guard cell plasma membrane. Carbonic anhydrases (CAs) contribute to this activation, but their contribution as distinct from CO_2_-H_2_CO_3_ catalysis remains controversial. Here we show that the β-carbonic anhydrase CA4 binds selectively with the guard-cell anion channel SLAC1 to enhance channel current. The interaction is CO_2_-dependent, but binding is mediated by amino acids distal from the CO_2_-binding site of CA4 and is separable from carbonic-anhydrase activity. CA4 mutants impaired in channel binding eliminate the CO_2_-sensitivity of SLAC1 in vivo and slow stomatal kinetics with a commensurate loss in water use efficiency. The findings demonstrate that CA4 contributes directly to the CO_2_-response mechanics regulating SLAC1 at near-ambient CO_2_ in guard cells and to stomatal kinetics in the plant.

## Abstract (web summary)

Separate from its catalytic activity, b-carbonic anhydrase 4 binds directly the Cl- channel SLAC1 in Arabidopsis guard cells, promoting SLAC1 activity and stomatal responsiveness to CO2, and facilitating plant growth.

## Introduction

Stomata are pores that occur within the epidermis of the leaves of land plants and are the principal route for CO_2_ diffusion from the atmosphere to the mesophyll for photosynthesis. They also facilitate water loss from the inner air space of the leaf to the atmosphere. This exchange of water for CO_2_ means that stomata must frequently balance the demand for CO_2_ in photosynthesis against the need to prevent the leaf from drying, thereby restricting photosynthesis especially when water delivery to the leaf is limited^1,2^. Stomata connect the global water and carbon cycles, affecting both, and are important in modelling for weather prediction^3,4^. Indeed, stomata lie at the center of the crises in fresh water availability and crop production that are projected over the next 20–30 years^5–7^.

The aperture of the stoma is controlled by the turgor of the guard cells that surround the pore. Guard cells respond to internal and external cues, notably changes in light, the partial pressure of CO_2_ in the atmosphere (pCO_2_), especially within the leaf (pC_i_), and atmospheric relative humidity^6,7^. They transport solutes, notably K^+^ salts, and water across the plasma membrane and tonoplast to adjust cell turgor^8,9^. Guard cell H^+^-ATPases extrude H^+^ to generate a pH difference and a membrane voltage, negative inside, that facilitates K^+^ uptake through K^+^ channels, in Arabidopsis principally KAT1^10–12^, and high-affinity K^+^ transport^13–15^. Stomata close when reduced H^+^-ATPase activity and Cl^-^ efflux combine to depolarize the plasma membrane thereby activating outward-rectifying K^+^ channels to promote K^+^, Cl^-^ and water loss from the guard cells.

A dominant pathway for Cl^-^ efflux is the SLAC1 anion channel. Its elimination in the *slac1* null mutant greatly slows stomatal closing^16,17^ as well as opening^18^. The SLAC1 channel is activated by elevated cytosolic-free [Ca^2+^] ([Ca^2+^]_i_)^19–21^, and is subject to phosphorylation directly by the kinases OST1 and CIPK23/CBL1^22–25^, and may be affected indirectly through interactions between the MPK12 and HT1 kinases^26,27^. Previous research showed that SLAC1 activity also depends on β-carbonic anydrases, notably the β-carbonic anydrase CA4^28,29^. These studies suggested that the channel responds to HCO_3_^-^ rather than dissolved CO_2_. However, they did not distinguish the action of CA4 on SLAC1 from its enzymatic activity in equilibrating CO_2_ with H_2_CO_3_ to generate HCO_3_^-^ in solution, and they have since drawn criticism for the use of non-physiological levels of inorganic carbon^7,19^. Thus, a question remains whether the CA is simply required for CO_2_ equilibration with H_2_CO_3_ to generate HCO_3_^-^ or has a direct role in regulating SLAC1. Furthermore, if CA4 does regulate SLAC1 activity, questions arise of how a CO_2_/HCO_3_^-^ signal is transmitted to SLAC1 and whether it depends on CA enzyme activity.

To address these questions, we examined SLAC1 binding with the predominant CAs of the guard cell to assess their specificity and the mechanistic connection to channel activity. Here we report that SLAC1 binds preferentially with CA4 and that CA4-SLAC1 interaction is a key factor in promoting SLAC1 activity at near-ambient pCO_2_. CA4-SLAC1 interaction shows a stoichiometric dependence on the CA4:SLAC1 ratio on heterologous expression, and it is suppressed by mutations that eliminate CA4 catalysis. However, CA4 catalysis and SLAC1 binding are separable. CA4 binding and its impact on SLAC1 activity depend on a motif distal from the catalytic site of CA4. Mutating residues within this motif suppresses CA4-SLAC1 interaction and SLAC1 current enhancement, but not CA enzyme activity. These mutations slow stomatal kinetics with CO_2_, and they reduce whole-plant water use efficiency (WUE) and growth when light, and hence pC_i_, vary over the day. We conclude that CA4 binding with the anion channel introduces a direct level of control by CO_2_/HCO_3_^-^ on SLAC1 activity.

## Results

### SLAC1 interacts selectively with β-carbonic anhydrase 4

Stomatal movement in Arabidopsis is affected by the null mutant of two β-carbonic anhydrases, CA1 and CA4, that localise to the chloroplast and to the cytosol and plasma membrane, respectively^29,30^. By contrast, the β-carbonic anhydrase CA3, normally present in the cytosol and expressed in guard cells^30^, has not been reported to affect stomata. We used the yeast mating-based split-ubiquitin screen (mbSUS) to test for interactions of the carbonic anhydrases with the Cl^-^ channel SLAC1. The mbSUS assay enables tests with full-length membrane proteins and yields growth of the diploid yeast on selective media only when the two halves of ubiquitin are brought together and reassemble through interaction of protein partners to which they are fused^31–33^.

**Fig. 1:**
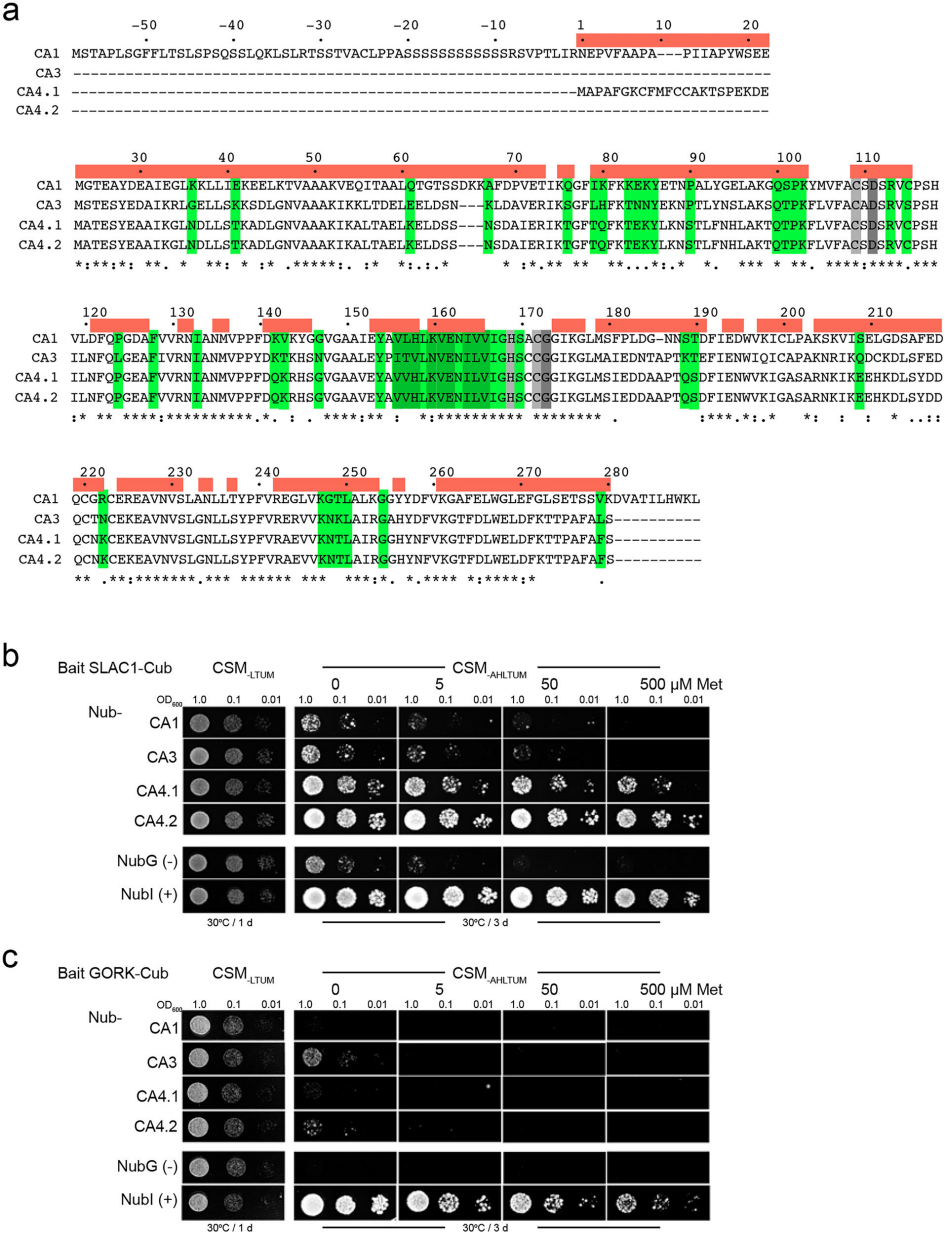
The β-carbonic anhydrase CA4 isoforms interact selectively with SLAC1 and not with the GORK K^+^ channel. **a** Alignment of Arabidopsis β-carbonic anhydrases CA1, CA3 and the CA4 isoforms CA4.1 and CA4.2. Sequence numbering is to CA4.1 with residue identity, charge conservation and similarities indicated below. Conserved residues that coordinate the Zn cofactor (light gray) and CO_2_/H_2_CO_3_ (dark gray) are indicated along with surface exposure (red bars, *above*). Residues in CA4.1 targeted for site mutations (green) were selected, in part, on the basis of surface exposure and variance from both CA1 and CA3. Yeast mating-based split-ubiquitin (mbSUS) assay for binding with SLAC1-Cub (**b**) and GORK-Cub (**c**) as baits with Nub-fusions of the β-carbonic anhydrases CA1, CA3, CA4.1 and CA4.2 as preys, including controls ([-], NubG; [+], NubI). One of five independent experiments, all yielding similar results. Yeast diploids dropped at 1.0, 0.1 and 0.01 OD_600_ spotted (*left to right*) on complete synthetic medium without Trp, Leu, Ura and Met (CSM_-LTUM_) to verify mating, on CSM without Trp, Leu, Ura, Ade, His and Met (CSM_-LTUMAH_) to verify adenine- and histidine-independent growth, and with Met additions as indicated to suppress bait expression as a test for interaction strength (strong interaction gives growth even on Met). Immunoblots are included in Supplemental Fig. 11.

The β-carbonic anhydrase CA4 occurs as two isoforms in vivo^30^: CA4.1 incorporates an N-terminal amphipathic sequence of 22 residues that appears to anchor it to the membrane, whereas CA4.2 lacks these residues (Fig. 1a). We observed growth (Fig. 1b) on selective media with diploid yeast expressing the full-length SLAC1-Cub bait with Nub-CA4.1 and Nub-CA4.2 as preys. Growth was recovered even on 500 μM Met that strongly reduces bait expression, indicating strong and specific interactions between SLAC1 and the two CA4 isoforms. Little growth was observed with Nub-CA1 and Nub-CA3, and neither showed growth on additions of 50 μM or 500 μM Met, indicating that these interactions were weak and non-specific. In each case, bait and prey expression were validated by immunoblot (Supplemental Fig. S11).

We carried out mbSUS assays also using as bait the outward-rectifying K^+^ channel GORK that, along with SLAC1, is coordinately regulated to facilitate solute loss for stomatal closure^7,8,34^. Again expression was validated by immunoblot (Supplemental Fig. S11). With the K^+^ channel as bait, no yeast growth was observed with any of the CAs when Met was present to test for specificity (Fig. 1c). Overall, the observations indicated a preferential interaction of SLAC1 with the CA4 isoforms.

To test whether the presence of both channel and CA might affect their distributions, we expressed SLAC1-GFP and the CAs as mCherry fusions transiently in tobacco leaf epidermis. As expected (Supplemental Fig. 1a), SLAC1 showed a peripheral distribution, consistent with its presence at the plasma membrane. Much the same distribution was observed for CA4.1 expressed alone and together with SLAC1. By contrast, CA3 and CA4.2, the latter lacking the amphipathic N-terminus (Fig. 1a), were evident in cytosolic strands and around nuclei both when expressed alone and together with SLAC1, indicating a cytosolic localisation.

Additionally, we carried out combined microsomal and two-phase separations followed by immunochemical analysis to localise CA4.1 and CA4.2 (Supplemental Fig. 1b). The primary antibody for the β-carbonic anhydrase did not distinguish between the two isoforms, but these are separable on the basis of their molecular weights. We generated a SLAC1 antibody using the N-terminal cytosolic domain of the channel (Supplemental Fig. 2). Fractionation analysis (Supplemental Fig. 1b) confirmed the presence of CA4.1 at the plasma membrane^35^ with a component evident in the inner membrane fraction that may reflect a traffic of the protein^36–38^. The results also showed that CA4.2 resides primarily in the cytosol, although a weak signal consistent with membrane-localised CA4.2 may be present and arise from dimerisation with CA4.1^39,40^ (below).

### CA4 mutations of the carbonic anhydrase catalytic domain lose SLAC1 binding

Plant β-carbonic anhydrases assemble as functional dimers and multimers that incorporate zinc cofactors for CO_2_ binding. The β-carbonic anhydrases are well-defined with crystal structures from bacteria, algae and angiosperms^39,40^, all conforming to a common α/β sheet structure with a cleft for binding of the substrates CO_2_ and H_2_O between subunits. Mutations of residues that coordinate the zinc cofactor and are necessary for CO_2_ binding within the cleft eliminate the enzymatic activity; these residues are highly-conserved (see Fig. 1a) across CAs^39,41^.

**Fig. 2:**
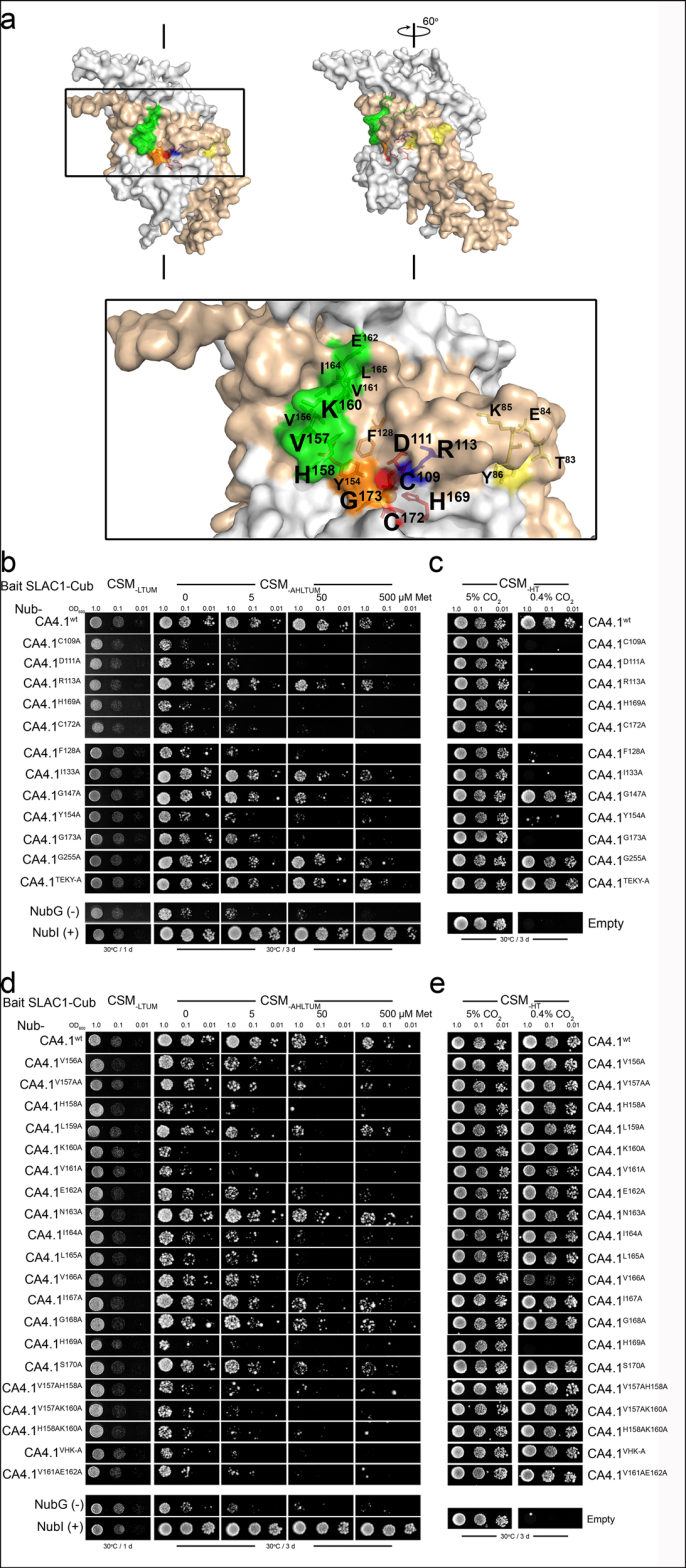
Residues needed for β-carbonic anhydrase CA4.1 binding with SLAC1 include, but are not limited to, those essential for catalytic activity. **a** Mapping of CA4.1 to the crystal structure of the *Pisum sativum* β-carbonic anhydrase^40^ with space-filled monomers of the dimer shaded in greys. Colour-coded are residues of the catalytic site C^109^, D^111^, H^169^ and C^172^ in red; the shielding residues F^128^, Y^154^ and G^173^ in orange; the SLAC1-binding motif V^156^, V^157^, H^158^, K^160^, V^161^, E^162^, I^164^, and L^165^ in green; R^113^ in blue; and the distal surface T^83^, E^84^, K^85^, and Y^86^ in yellow. Colour-coded residues are also shown in stick representations with labels. Internal sphere represents the zinc cofactor within the CO_2_-binding pocket. **b** Yeast mating-based split-ubiquitin (mbSUS) assay for binding of SLAC1-Cub as bait with Nub-CA4.1 wild-type (CA4.1 ^wt^) and single-site, Ala mutations of core (CA4.1^C109A^, CA4.1^D111A^, CA4.1^H169A^, CA4.1^C172A^) and associated residues (CA4.1^F128A^, CA4.1^I133A^ CA4.1^Y154A^, CA4.1^G173A^) essential for CA activity. Additional proximal site mutants (CA4.1^R113A^, CA4.1^G147A^, CA4.1^G255A^) and the quadruple mutant CA4.1^TEKY-A^ included with controls ([-], NubG; [+], NubI). One of four independent experiments, all yielding similar results. Yeast diploids dropped at 1.0, 0.1 and 0.01 OD_600_ spotted (*left to right*) on complete synthetic medium without Trp, Leu, Ura and Met (CSM_-LTUM_) to verify mating, on CSM without Trp, Leu, Ura, Ade, His and Met (CSM_-LTUMAH_) to verify adenine- and histidine-independent growth, and with Met additions as indicated to suppress bait expression. **c** Complementation with the same mutations (**b**) of the yeast *ΔNCE103* mutant that normally requires 5% CO_2_ shows growth rescue at ambient CO_2_ only with CA4.1 ^wt^, CA4.1^G147A^, CA4.1^G255A^ and the quadruple mutant CA4.1^TEKY-A^. The *ΔNCE103* mutant background (Empty) and complemented yeast plated on synthetic media minus histidine and threonine (CSM_-HT_) to verify the background and grown under ambient (0.4%) and 5% CO_2_. **d** Yeast mbSUS assay for binding of SLAC1-Cub as bait with Nub-CA4.1 wild-type (CA4.1 ^wt^), single-site, Ala mutations of surface-exposed residues spanning the V^156^VHxKVExIL SLAC1-binding motif, and selected double and triple mutations within the motif. Controls ([-], NubG; [+], NubI) included for reference. One of four independent experiments, all yielding similar results. Yeast diploids dropped at 1.0, 0.1 and 0.01 OD_600_ spotted (*left to right*) on complete synthetic medium without Trp, Leu, Ura and Met (CSM_-LTUM_) to verify mating, on CSM without Trp, Leu, Ura, Ade, His and Met (CSM_-LTUMAH_) to verify adenine- and histidine-independent growth, and with Met additions as indicated to suppress bait expression. **e** Complementation with the same mutations (d) of the yeast *ΔNCE103* mutant that normally requires 5% CO_2_ shows growth rescue at ambient CO_2_ with each of the motif mutations. The *ΔNCE103* mutant background (Empty) and complemented yeast plated on synthetic media minus histidine and threonine (CSM_-HT_) to verify the background and grown under ambient (0.4%) and 5% CO_2_. Immunoblots are included in Supplemental Fig. 11.

We wanted to know if CA activity is important for its binding with SLAC1. Initial experiments focused therefore on binding within the CA4.1 backbone using the *Pisum sativum* crystal structure as a guide^40^ for site mutagenesis (Fig. 2a). We generated mutations with Ala substitutions for residues C^109^, H^169^ and C^172^ that bind the zinc cofactor, for residues D^111^ and G^173^ that contribute to CO_2_ coordination^41^ and, as a control, the adjacent R^113^ residue (Fig. 2b). As CO_2_ binding occurs behind a hydrophobic fold of F^128^, I^133^ and the surface-exposed Y^154^, we targeted these residues for Ala substitution along with residues G^147^ and G^255^ that are not surface-exposed and residues T^83^, E^84^, K^85^ and Y^86^ that situate on the surface of CA4.1 roughly 20 Å from the catalytic site (Fig. 2a).

In each of these experiments (Fig. 2b and Supplemental Fig. 3a), we found that mutations of any of the residues coordinating the zinc cofactor and CO_2_, as well as residues F^128^ and Y^154^ of the hydrophobic fold, resulted in a loss of interaction with SLAC1 in mbSUS assays; by contrast, the R^113A^ mutant, the distal residue substitutions I^133A^, G^147A^, and G^255A^, and the quadruple Ala substitution of T^83A^E^84A^K^85A^Y^86A^ (CA4.1^TEKY-A^) did not affect yeast growth. The CA4.1 isoform also includes, within the N-terminus, three Cys residues that could serve as palmitoylation sites for membrane anchoring. However, the C^8S^C^12S^C^13S^ triple mutant (CA4.1^CTS^), when used as prey with the SLAC1 bait in mbSUS assays, showed no difference in yeast growth compared to the wild-type isoform CA4.1 ^wt^ (Supplemental Fig. 3a), indicating that these sites are not important for SLAC1 binding, a conclusion also evident from interaction with CA4.2 that lacks this N-terminal extension.

To verify the effects of catalytic site mutations on CA activity, we made use of the CA-free null, *ΔNCE103* yeast mutant^42^, complementing with each of the CA4.1 mutations. The *ΔNCE103* strain fails to grow unless maintained under 50,000 μbar (5%) CO_2_. Of the complemented *ΔNCE103* yeast, we found that only those carrying the CA4.1 wild-type (CA4.1 ^wt^), the G^147A^, G^255A^, and T^83A^E^84A^K^85A^Y^86A^ substitutions were able to grow at the ambient 400 μbar CO_2_ (Fig. 2c). Thus, we concluded that mutations of CA4.1 associated with the catalytic site and buried within the CA structure^40^ nonetheless prevent binding with SLAC1. However, mutants R^113A^ and I^133A^ that interact with SLAC1 suppressed catalytic activity, demonstrating that binding and catalytic activity are separable and raising a question about the identify of the SLAC1 binding site.

### Identifying the SLAC1 binding motif

Alignment of CA4.1 and CA4.2 with CA1 and CA3 highlighted a number of residues away from from the N-terminus that differed in both CA1 and CA3 and might be important for SLAC1 binding. Mapping these residues to the crystal structure of the *Pisum sativum* β-carbonic anhydrase^39,40^ (Figs. 1a and 2a) indicated that a majority reside at the protein surface and might therefore be candidates for interaction with the channel. An appreciable density of these residues situated around an exposed cleft central to the CA structure and either side of conserved residues nearby the three residues H^169^, C^172^ and G^173^ that are critical for enzyme activity. We carried out alanine-scanning mutagenesis at each of these sites, along with more distal, surface-exposed residues that differed from both CA1 and CA3, testing the impacts on SLAC1 interaction by mbSUS assay and on CA catalytic activity by rescue of *ΔNCE103*-complemented yeast growth at ambient CO_2_, as before.

mbSUS assays identified residues between V^156^ and H^169^ that affected SLAC1-CA4 interaction. Each of the single residue substitutions - notably V^157A^, H^158A^, and K^160A^ - greatly reduced or eliminated yeast growth, even under mild bait suppression; yet, with the exception of V^166A^, each mutation yielded growth at ambient CO_2_ on complementing *ΔNCE103* yeast (Fig. 2d,e). Similarly, combined mutations of V^157A^, H^158A^, and K^160A^ eliminated yeast growth under selection in mbSUS assays but rescued *ΔNCE103* yeast growth at ambient CO_2_. Of residues at the other, unique and surface-exposed sites, none suppressed SLAC1 interaction or catalytic activity based on the yeast growth assays (Supplemental Fig. 3).

In support of these findings, we carried out co-immunoprecipitation experiments using transient expression in the *ca1ca4* mutant Arabidopsis background with the GFP-tagged channel as bait and with mCherry-tagged CA forms. Elution of SLAC1 bound to GFP-trap resin^43^ recovered the channel together with CA4.1 ^wt^ and CA4.1^R113A^, but little or no recovery was observed with CA4.1^D111A^, CA4.1^VHK-A^ and CA4.1^C172A^, nor with CA3 as a negative control (Supplemental Fig. 4). Thus, we concluded that SLAC1 binds directly with CA4.1 and the sequence V^157^HxK marks the center of a key motif for SLAC1 binding independent of CA4 carbonic anhydrase activity.

**Fig. 3:**
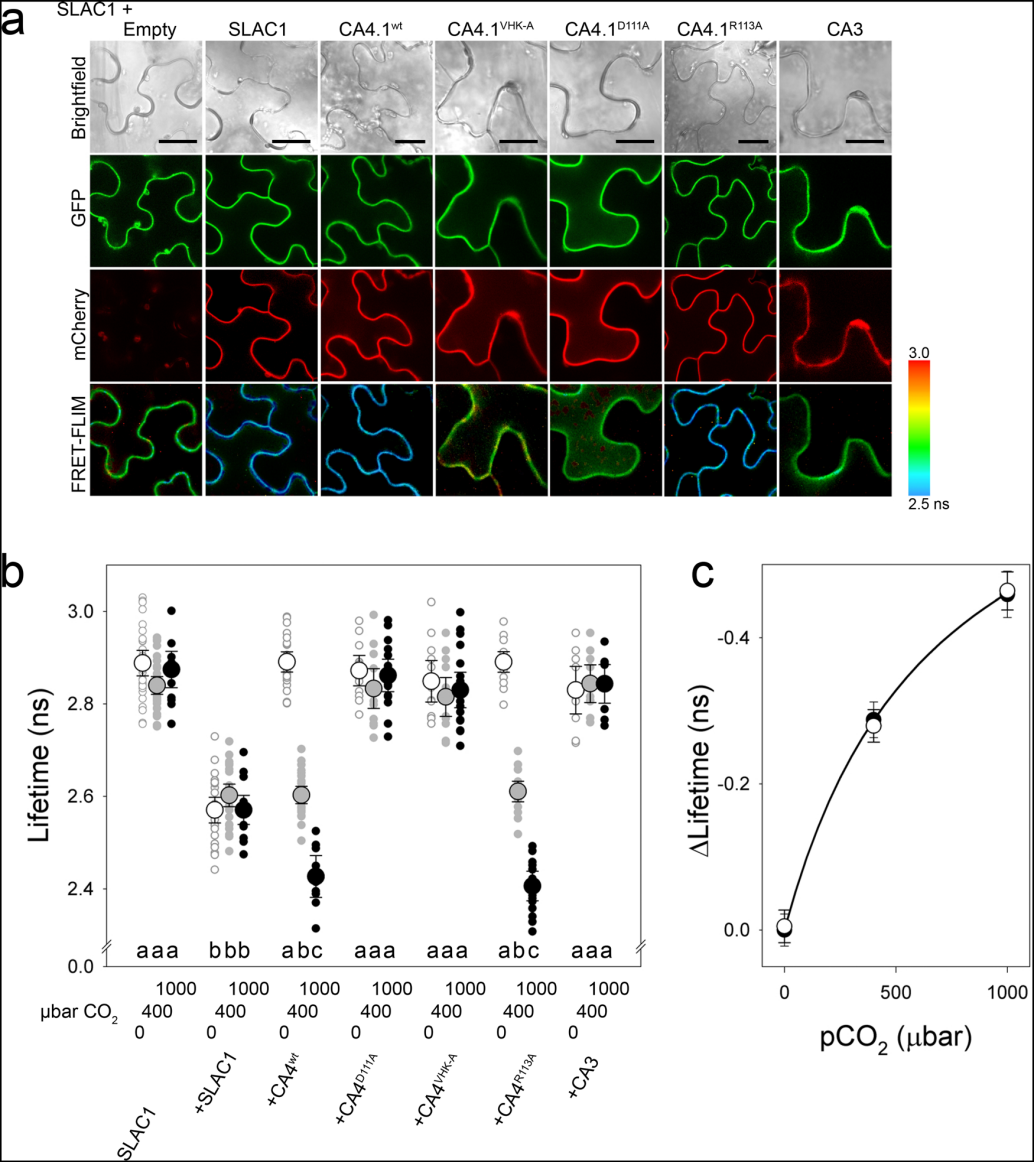
Fluorescence resonance energy transfer lifetime imaging (FRET-FLIM) of SLAC1 interaction with CA4.1. **a** Representative image analysis of SLAC1-GFP with mCherry fusions of SLAC1 and CA3 as positive and negative controls, of CA4.1 ^wt^, and of the mutants CA4.1^VHK-A^, CA4.1^D111A^ and CA4.1^R113A^ at 400 μbar CO_2_ expressed in tobacco leaf epidermis. Fluorescence lifetimes colour-coded (scale*, lower right*; scale bars, 20 μm). **b** Individual data and means ± SEM from >10 independent transformations at each of 0, 400 and 1000 μbar CO_2_ show decreasing fluorescence lifetimes with increasing pCO_2_, indicating SLAC1 interaction with CA4.1 ^wt^ and CA4.1^R113A^ but not with mutants suppressing SLAC1-CA4.1 binding. Fluorescence lifetime decrease evident for SLAC1 self-interaction (positive control) was independent of pCO_2_. Significant differences at P < 0.02 are indicated by lettering. **c** Means ± SEM for CA4.1 ^wt^ (●) and CA4.1^R113A^ () from (**b**) replotted as the inverse complement (= τ_f(i)_-τ_f(0)_, where τ_f(i)_ and τ_f(0)_ are the fluorescence lifetimes for pCO_2_ = *i* and 0, respectively) and fitted by non-linear least-squares to a hyperbolic function (*solid line*) gave an apparent K_1/2_ of 679 ± 40 μbar CO_2_ for interaction.

To validate CA4-SLAC1 interactions in vivo, we used fluorescence lifetime imaging microscopy (FRET-FLIM) after transiently transforming tobacco leaf epidermis using the 2in1 vector system that ensures equal genetic loads and reduces variability in protein-protein interaction analyses^44^. We tagged the CA4 constructs with mCherry and SLAC1 with GFP. As SLAC1 assembles in trimers at the membrane^25,45^, we also expressed SLAC1-GFP together with SLAC1-mCherry as a positive control. The FRET-FLIM analysis (Fig. 3a,b) showed a highly significant reduction in fluorescence lifetime for SLAC1-GFP on coexpressing mCherry-CA4.1 ^wt^, comparable to that observed on co-expressing the SLAC1-mCherry positive control. By contrast, co-expressing SLAC1-GFP with CA4.1 mutations that suppressed interaction - either the CA4.1^D111A^ or the V^157A^H^158A^K^160A^ triple Ala substitution of the SLAC1-binding motif, hereafter designated CA4.1^VHK-A^ - yielded lifetimes similar to that of the donor alone. As in the mbSUS and co-IP assays, the CA4.1^R113A^ mutant that lacks CA catalytic activity retained interaction in vivo, as indicated by the reduced fluorescence lifetime.

We extended the FRET-FLIM experiments to test for a dependence of the SLAC1-CA4 interaction on pCO_2_. Measurements were carried out on transformed tobacco leaves in 0 and 1000 μbar CO_2_ as well as at ambient (400 μbar) CO_2_. The FRET-FLIM analysis (Fig. 3a,b) showed further reductions in fluorescent lifetimes with 1000 μbar CO_2_ for SLAC1-GFP on coexpressing with the mCherry-tagged CA4.1 ^wt^ and CA4.1^R113A^ mutant, but not with the CA4.1^VHK-A^ and CA4.1^D111A^ mutants (Fig. 3b). On co-expressing mCherry-tagged CA4.1 ^wt^ and CA4.1^R113A^, we also observed little or no decline in fluorescent lifetime compared with the negative controls when measurements were carried out in 0 μbar CO_2_ (Fig. 3b). Converting these data to their complements (Fig. 3c) yielded an hyperbolic relation with an apparent midpoint of 679 ± 40 μbar CO_2_. Thus, we concluded SLAC1 interacts directly with CA4.1 at the plasma membrane, that this interaction is subject to the binding motif centered on V^157^HxK as well as to mutations within the CA catalytic site, and that the interaction occurs with an apparent K_1/2_ broadly consistent with the action of pCO_2_ on SLAC1 and stomata in vivo^16,46^.

### Binding-impaired CA4 fails to enhance SLAC1 activity

SLAC1 yields a current under voltage clamp, both in vivo and on heterologous expression in *Xenopus* oocytes, that activates at positive voltages and deactivates slowly, yielding a near-linear current-voltage (IV) curve over short times when the membrane is stepped negative^16,21,47,48^. SLAC1 activity was reported previously to be enhanced in oocytes co-expressing CA4 and the aquaporin PIP2;1, proposed to interact with the CA^28^. Evidence for any specificity in binding was missing, however, and the extreme concentrations of HCO_3_^-^ needed to activate SLAC1 (roughly equivalent to 100 times atmospheric CO_2_) discount any physiological significance. Indeed, to date there remains little evidence pertinent to CA4 action on the anion channel.

**Fig. 4:**
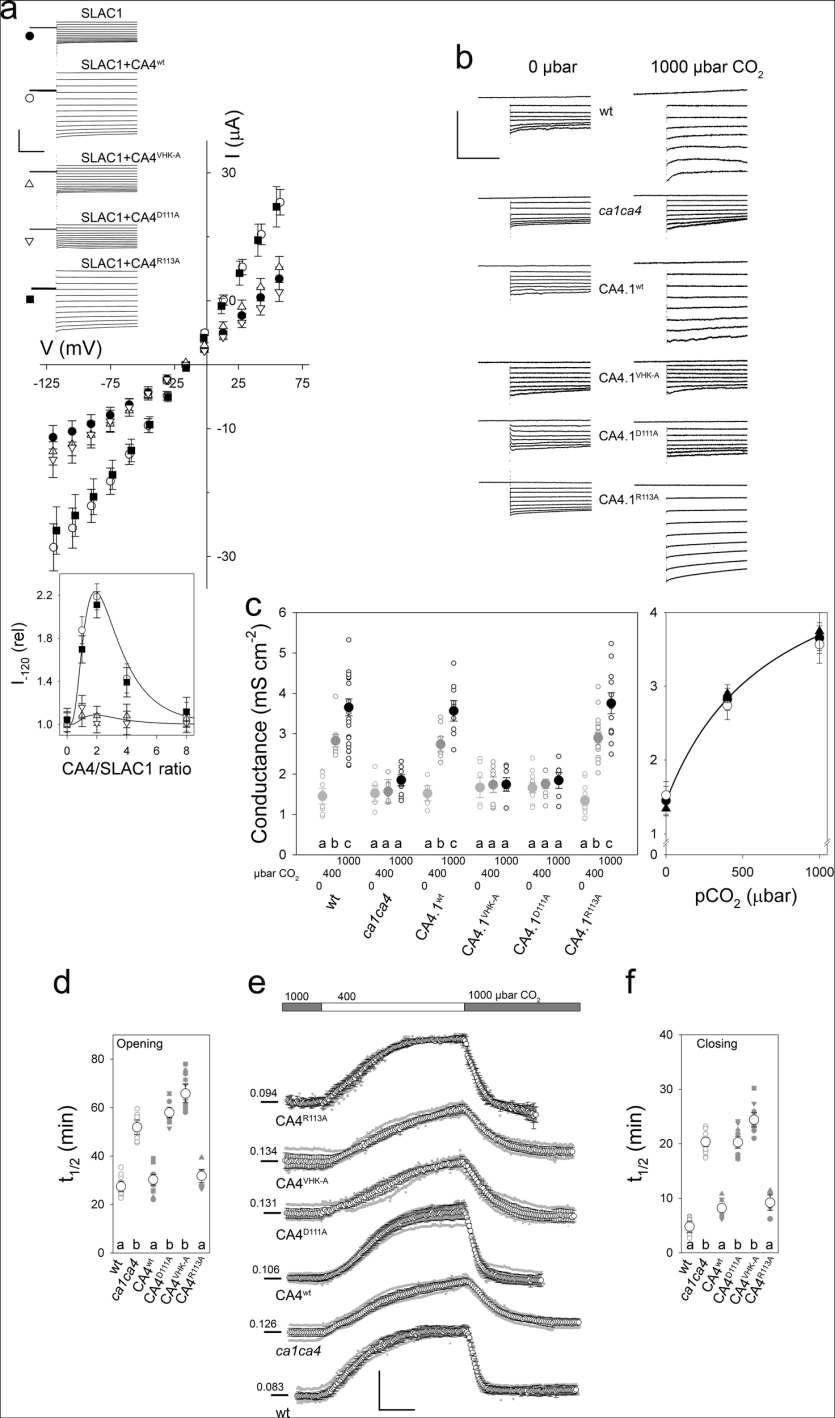
Binding of β-carbonic anhydrase CA4.1 promotes the SLAC1 anion current and accelerates stomatal closing and opening. **a** Representative current traces (insets, *above left*) and mean steady-state current-voltage (IV) curves recorded under voltage clamp from oocytes expressing SLAC1 alone (●), and with CA4.1 ^wt^ (), CA4.1^VHK-A^ (Δ), CA4.1^D111A^ (∇) and CA4.1^R113A^ (■). All experiments included co-expression of CBL1 and kinase CIPK23 prerequisite for SLAC1 activity. Scale bars: horizontal, 1 s; vertical, 30 µA. IV curves are means ± SEM of n≥8 independent experiments, cross-referenced to the traces by symbol. Mean current at −120 mV on co-expression with CA4.1 ^wt^ and CA4.1^VHK-A^ (inset, *below*) shows a maximum effect with molar ratios of 2:1 (CA:SLAC1). (b,c) Representative current traces (**b**) and mean SLAC1 conductances (**c**) for the channel in Arabidopsis guard cells of wild-type (wt) and *ca1ca4* mutant (*ca1ca4*) plants and in the *ca1ca4* mutant stably expressing CA4.1 ^wt^, CA4.1^VHK-A^, CA4.1^D111A^ and CA4.1^R113A^ as indicated. Scale bar (**b**): horizontal, 1 s; vertical, 400 μA cm^−2^. Data points in (**c**) are from independent transformants (*small open symbols*; n > 7 for each genotype, *left*) and their means ± SEM (*filled symbols*). Measurements in CO_2_-equilbrated buffers showed strong enhancement with 1000 μbar CO_2_ in the wild-type, CA4.1^wt^-and CA4.1^R113A^-complemented lines but not in the in the *ca1ca4* mutant background alone or expressing CA4.1^VHK-A^ and CA4.1^D111A^. Non-linear least-squares fitting of the means ± SEM for the wild-type (●), CA4.1^wt^- () and CA4.1^R113A^-complemented (▲) lines to a hyperbolic function with offset (*right*) yielded apparent K_1/2_ values of 692 ± 94, 701 ± 81 and 651 ± 104 μbar CO_2_, respectively. Fitting to the wild-type data shown (*solid line*) for visual reference. Stomatal conductances recorded on stepping between 400 and 1000 μbar CO_2_, with the corresponding halftimes for opening (**d**) and closing (**f**). Scale bar (**e**): horizontal, 30 min; vertical, 100 mmol m^−2^s^−1^. Data points in (**d**-**f**) are from independent transformants (*small symbols*; *n* > 7 for each genotype) and their means ± SEM (*large open symbols*). Significant differences (**d**, **f**) at *P* < 0.02 are indicated by lettering. Immunoblots (**a**-**f**) are included in Supplemental Fig. 11.

We expressed SLAC1 in oocytes with the protein kinase CIPK23 and its partner, calcineurin-binding protein CBL1 for phosphorylation needed for channel activity^16,28,47^. SLAC1 was co-expressed also with CA4.1 in stoichometric ratios relative to the anion channel, and SLAC1 and CA4.1 expression levels were validated by immunoblot of the oocytes used for electrical recordings (Supplemental Fig. S11). Following activation at +30 mV, voltage clamp measurements yielded typical, quasi-linear characteristics of the active channel that were enhanced roughly 2-fold on co-expressing CA4.1 ^wt^ in 1:1, and further enhancement was recovered using 2:1 ratios of the CA with SLAC1 (Fig. 4a). Higher ratios of CA4.1 ^wt^:SLAC1 showed a loss in current enhancement (Fig. 4a, *inset*), indicating a biphasic action of the carbonic anhydrase on the channel, possibly through assembly of higher-order CA complexes^39,40^ and masking of the binding sites.

We repeated these measurements with the non-binding CA4.1^VHK-A^ and CA4.1^D111A^ mutants, the latter also impaired in catalytic activity, and with the CA4.1^R113A^ mutant that retains binding but not catalytic activity. Both the CA4.1^VHK-A^ and CA4.1^D111A^ mutants failed to enhance the current, regardless of the CA4.1:SLAC1 ratio, whereas the CA4.1^R113A^ mutant that retains binding but not CA activity enhanced the SLAC1 current (Fig. 4a). By contrast with guard cells (below), efforts in oocytes to identify a dependence of the current over a range of CO_2_ partial pressures were not successful, suggesting that one or more additional components normally present in the plant and needed for dynamic regulation by CO_2_ were absent in the oocyte assays (see **Discussion**). Nonetheless, the results show that mutations suppressing CA4-SLAC1 interaction fail to promote SLAC1 channel activity in a manner separable from catalytic activity.

### CA4 binding is necessary for CO_2_-enhanced SLAC1 current and stomatal dynamics in vivo

We next asked whether CA4.1 mutations that impair SLAC1 binding also affect channel activity and stomatal function in vivo. We generated, in the Arabidopsis *ca1ca4* null background, stable transformants of the *CA4.1*^*VHK-A*^ and *CA4.1*^*D111A*^ mutations that suppress CA4.1-SLAC1 interaction, of the *CA4.1*^*R113A*^ mutation that retains binding but blocks CA catalytic activity, and of *CA4.1 *^*wt*^ as a control. Expression was driven by the native *pCA4* promoter, and transformants were selected for growth on hygromycin and thereafter for immunoreactivity (see Supplemental Fig. S11). To avoid potential silencing with channel expression^49^, experiments were carried out on T1 generation plants, so that each plant corresponded to an independent transformant, and on at least two independent T2 generation lines with each plant carried through electrophysiology and gas exchange studies. We observed no systematic differences between transformants, and the results from the individual transformants are presented along with the pooled statistics.

We recorded SLAC1 currents under voltage clamp^50^ from intact guard cells in epidermal peels of the transformed plant lines and, as controls, from the wild-type and *ca1ca4* null mutant backgrounds. Recordings were carried out with CsCl-filled microelectrodes and with CsCl and tetraethylammonium chloride (TEA-Cl) in the bath to eliminate the background of K^+^ currents^21,48^. Continuous superfusion was maintained during recordings with bath solutions pre-equilibrated with 0, 400 and 1000 μbar CO_2_. As in the oocytes, guard cells of wild-type Arabidopsis yielded anion currents that deactivated only slowly on stepping the voltage negative from +30 mV. Furthermore, exposures to solution pre-equilibrated with 400 μbar CO_2_, and especially with 1000 μbar CO_2_, showed enhanced anion currents (Fig. 4b).

We quantified the currents using the instantaneous conductances recorded following 10-s activation steps at +30 mV to voltages between +30 and −180 mV (Fig. 4c). In guard cells of wild-type Arabidopsis, these currents yielded mean conductances of 1.41 ± 0.16 mS cm^−2^ under 0 μbar CO_2_ with a mean 1.9-fold enhancement to 2.58 ± 0.18 mS cm^−2^ under 400 μbar CO_2_ and a 2.8-fold enhancement to 3.96 ± 0.21 mS cm^−2^ under 1000 μbar CO_2_. Similar currents were recorded from guard cells of *ca1ca4* mutant plants complemented with *CA4.1* ^*wt*^ and with *CA4.1*^*R113A*^ but current enhancement was absent in guard cells of the *ca1ca4* mutant background. Recordings from guard cells of the *ca1ca4* mutant complemented with the non-interacting *CA4.1*^*VHK-A*^ mutation and with the non-interacting and catalytically-impaired *CA4.1*^*D111A*^ mutation failed to show any increase in anion current conductance, even under 1000 μbar CO_2_. Plotting the conductances from the wild-type Arabidopsis, the *CA4.1*^*wt*^- and *CA4.1*^*R113A*^-complemented plants (Fig. 4c, *right*) yielded hyperbolic relations with apparent midpoints of 692 ± 94, 701 ± 81 and 651 ± 104 μbar CO_2_, respectively. These results indicate an action with apparent K_1/2_ values similar to those for SLAC1-CA4 interaction in vivo (Fig. 3). Most important, they show that CA binding with SLAC1 is necessary for the increased SLAC1 current with CO_2_, separable from CA catalytic activity, and that the SLAC1-CA4 response is graduated over the normal physiological range for pCO_2_/HCO_3_^-^.

Simulations using the OnGuard3 modelling platform^19,51^ (Supplementary Data 2), predicted that increasing the apparent K_d_ for SLAC1 activation by HCO_3_^-^ by a factor of ten - so reducing the CO_2_ sensitivity of the channel - should reduce Cl^-^ efflux from the guard cells, slow stomatal closure and *g*_*s*_ relaxations with steps to 1000 μbar CO_2_, and impair the overall efficiency in water use by the plant (Supplemental Figs. 5 and 6). They also predicted a slowing of stomatal opening and recovery in *g*_*s*_ with steps back to 400 μbar CO_2_, much as was predicted and demonstrated experimentally for the *slac1* null mutant^18^. To test these predictions, we first recorded the timecourse of changes *g*_*s*_ using the same plants employed for electrophysiology (above). Experiments incorporated transitions between 400 and 1000 μbar CO_2_ under a constant 200 μmol m^−2^s^−1^ of photosynthetically-active radiation (PAR) after preconditioning with 200 μmol m^−2^s^−1^ PAR. The results (Fig. 4d-f) confirmed highly-significant increases in the halftimes for stomatal closure of 2.6- and 3-fold for CA4.1^D111A^ and CA4.1^VHK-A^, respectively, on transit to 1000 μbar CO_2_ when compared to the CA4.1^R113A^- and CA4.1^wt^-complemented *ca1ca4* double mutant. They also showed a corresponding increase in the halftimes for stomatal opening of 1.9- and 2.3-fold on returning to 400 μbar CO_2_.

### CA4-SLAC1 binding-impaired stomata alter plant growth

Plant performance is commonly quantified in relation to water use efficiency (WUE), that is the amount of dry matter produced per unit water transpired^2,52^. Light affects WUE through the carbon demand for photosynthesis and the transpiration it entails. In the natural environment, daylight often fluctuates, for example as clouds pass by. While photosynthetic capacity follows the energy input of light, stomata are generally much slower to respond to changes in light and, hence pC_i_. This mismatch between stomatal kinetics and photosynthetic capacity can lead to a limitation of photosynthesis by stomata when light intensity rises, and it can lead to water loss without corresponding gains in photosynthetic carbon assimilation when light intensity falls^2^. Thus, we reasoned that plants expressing the non-interacting CA4.1^VHK-A^ and CA4.1^D111A^ forms might show reduced WUE, assimilation and biomass, by comparison with the *CA4.1*^*R113A*^*-* and *CA4.1*^*wt*^-complemented lines, when grown under fluctuating light that drives concommitant fluctuations in pC_i_.

We used plants, selected as described above, and compared growth between the wild-type, the *ca1ca4* null mutant, and the *ca1ca4* lines complemented with *CA4.1 *^*wt*^, *CA4.1*^*D111A*^*, CA4.1*^*VHK-A*^, and *CA4.1*^*R113A*^ under control of the native *pCA4* promoter. All lines were grown with plants divided between two light regimes, one of a fixed daylight intensity of 140 μmol m^−2^s^−1^ PAR and the second of same daylight period and total fluence but with light varying at 20-min intervals between 40 and 240 μmol m^−2^s^−1^ PAR. The 20-min intervals approximated the time normally required for stomatal closing and roughly half of that for opening, thus exposing any appreciable differences in growth arising from impaired stomatal kinetics. Additionally, the lines within each light regime were divided for growth under 200, 400 and 1000 μbar CO_2_, and water was restricted to impose a drought stress by maintaining soil moisture at 10 ± 5% throughout the 5-week period of growth.

**Fig. 5:**
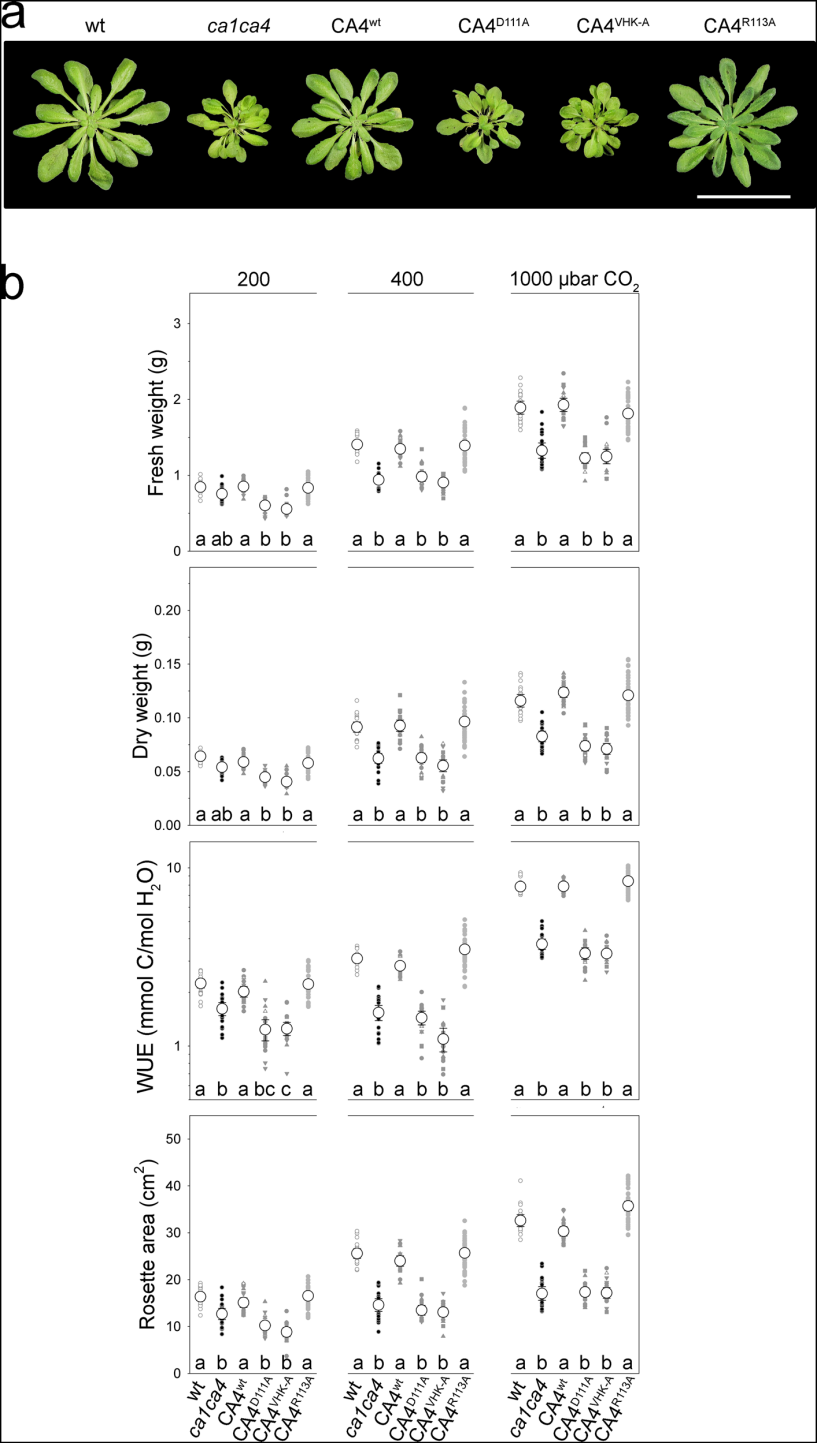
Impairing β-carbonic anhydrase CA4.1-SLAC1 binding leads to reduced growth and water use efficiency (WUE) under fluctuating daylight. **a** Representative rosettes of wild-type Arabidopsis (wt), the *ca1ca4* mutant background, and plants of the *ca1ca4* background complemented with *CA4.1 *^*wt*^, *CA4.1*^*VHK-A*^, *CA4.1*^*D111A*^ and *CA4.1*^*R113A*^ after 5 wk growth under fluctuating daylight at 400 μbar CO_2_. Scale: 5 cm. **b** Analysis of plant growth under fluctuating daylight with 200, 400 and 1000 μbar CO_2_ as indicated. Data are (*top to bottom*) fresh weight, dry weight, WUE and rosette area. Data points (*n* ≥ 10) for each genotype are independent plants and transformants (*small symbols*) and corresponding means ± SEM (*large open symbols*). Significant differences at *P *< 0.02 within each CO_2_ treatment are indicated by lettering. Note that WUE values are plotted on a logarithmic scale.

We found (Fig. 5 and Supplemental Fig. S7) that rosette areas, fresh and dry weights, and WUE of all plant lines increased with pCO_2_, and growth at any one pCO_2_ under fluctuating light was reduced by comparison with constant daylight conditions. Fluctuating light augmented differences between the lines especially at higher pCO_2_ when much greater variations in pC_i_ would be expected as stomata open and close. The *ca1ca4* mutant background showed reductions in every parameter when compared to wild-type plants. Of the lines complementing the *ca1ca4* background, plants expressing *CA4.1 *^*wt*^ and *CA4.1*^*R113A*^ were statistically indistinguishable from the wild-type control. However, plants expressing *CA4.1*^*D111A*^ and *CA4.1*^*VHK-A*^ showed reduced fresh and dry weights, rosette areas and WUE that were similar to those of the *ca1ca4* background, even though the CA4.1^VHK-A^ protein retains CA catalytic activity.

Our use of the native *pCA4* promoter to complement the *ca1ca4* null mutant, and the inclusion of a catalytically-functional, non-interacting β-carbonic anhydrase, suggested that any effects of CA4.1 complementations should arise from SLAC1 activity in the guard cells. Although homologous anion channels are expressed in the root and affect Cl^-^ and NO_3_^-^ balance in the plant^53^, SLAC1 is expressed primarily in the guard cells. However, CA4 is expressed in several vegetative tissues, including the root epidermis^54^. Thus, as a check against secondary effects associated with mineral nutrition, we grew plants of the wild type, *ca1ca4* mutant, and *ca1ca4* mutant complemented with *CA4.1 *^*wt*^*, CA4.1*^*D111A*^, and *CA4.1*^*VHK-A*^ on minimal salts media^55^ that included 10 mM Cl^-^ and 1 mM NO_3_^-^. No significant differences were observed between lines either in Cl^-^ and NO_3_^-^ content (Supplemental Fig. S8), suggesting that mutations affecting either SLAC1 activity or CA4 catalysis had no substantive impact on mineral balance.

Finally, we measured photosynthetic assimilation (Supplemental Fig. S9) under saturating 600 μmol m^−2^s^−1^ PAR with maximum steady-state *g*_*s*_. Over the physiological range of internal CO_2_ concentrations, similar rates were recorded in the CA4.1^VHK-A^- and CA4.1^D111A^-expressing plants as in wild-type Arabidopsis, the CA4.1^wt^- and CA4.1^R113A^-expressing plants. These results indicate that other CAs are sufficient to support photosynthesis and that any effects on biomass and WUE did not arise from differences in photosynthetic capacity. Instead, the phenotypic differences between transgenic lines are most simply understood to arise from their impacts on stomatal dynamics. Indeed, mutations affecting CA4.1-SLAC1 binding, at 400 μbar CO_2_, translated to a highly significant decline in WUE of 2.6- and 2-fold in the *CA4.1*^*VHK-A*^- and *CA4.1*^*D111A*^-complemented plants, respectively, when compared to the *CA4.1*^*wt*^-and *CA4.1*^*R113A*^-complemented plants (Fig. 5). Thus, complementing with *CA4.1* mutated to affect its binding with SLAC1 slowed stomatal kinetics independent of any effects of the β-carbonic anhydrase on CO_2_-H_2_CO_3_ equilibration and photosynthesis.

## Discussion

The SLAC1 anion channel is expressed primarily in guard cells and plays an important role as a dominant pathway^16,17^ for Cl^-^ efflux during stomatal closure^8,56^. SLAC1 also affects stomatal opening through the metabolic and signalling networks of the guard cell that, in the *slac1* mutant, otherwise suppress inward-rectifying K^+^ channel activities^18^. While [Ca^2+^]_i_ elevation and phosphorylation are important for SLAC1 activity^19–21,23,24^, much interest has centered on the actions of CO_2_ in promoting the current. Mutant analysis indicated roles for the chloroplastic and plasma membrane-localised β-carbonic anydrases CA1 and CA4, respectively^29^, identifying HCO_3_^-^ as a plausible ligand^57^. Additional studies suggested that an interaction of CA4 with the aquaporin PIP2;1 in oocytes facilitates SLAC1 activity^28^, although the findings have yet to be validated in the plant. Nonetheless, these and related studies do not separate any regulatory impacts of the CAs from their catalytic activities in equilibrating CO_2_ with H_2_CO_3_ and forming HCO_3_^-^. The studies have since been challenged also for the exceptionally high levels of HCO_3_^-^ needed to evoke a response in the SLAC1 current^7,19^.

Our findings put to rest the question of SLAC1 regulation and the catalytic activities of the guard cell CAs. We observed that the cytosolic and plasma membrane-associated CA4 binds selectively with SLAC1 to promote the anion channel current at near-ambient pCO_2_. CA4 interaction with SLAC1 was sensitive to mutations that eliminate substrate and zinc cofactor binding essential for catalytic activity; however, proximal mutations that eliminated carbonic anhydrase activity did not affect SLAC1 binding or enhancement of the channel activity. Furthermore, SLAC1 binding was blocked by mutations within a linear motif centered on residues V^157^HxK that did not affect the catalytic activity of CA4, and the same mutations suppressed SLAC1 current enhancement and slowed stomatal dynamics in response to near-ambient changes in pCO_2_. The effects on stomatal dynamics translated to reduced WUE and carbon assimilation in the intact plant, notably under fluctuating light that exposes phenotypes dependent on the speed of stomatal movements. These findings support the idea of CA4 as a key factor regulating SLAC1 with changes in pCO_2_, and they indicate a mechanism that acts through direct interaction of the CA with the anion channel. The findings will enable work to resolve the structural mechanics of CA binding with SLAC1, and they open the door to further research focused on ‘tuning’ the gating of these channels to improve WUE and photosynthetic yields.

### CO_2_ response factor or catalytic converter

For any CO_2_-dependent process, separating CA-mediated catalysis from a regulatory function that is effected through protein binding cannot be addressed using CA null mutants, such as undertaken in the past^29,57^. Even tampering with the catalytic site through single-residue exchanges is problematic. We found that substituting the residues for zinc cofactor binding and substitutions of the CO_2_- and H_2_O-coordinating residues abolished SLAC1 binding as well as catalytic activity (Fig. 2). These observations imply that catalytic site blockers such as acetazolamide^39^ are equally likely to interfere with SLAC1 binding and therefore cannot be used to distinguish between any role for the CA in SLAC1 regulation and its catalytic role in CO_2_-H_2_CO_3_ equilibration.

Alignment of CA4 with the non-binding CA1 and CA3 uncovered a subset of divergent residues, mutations of which prevented CA4-SLAC1 interaction without impacting on CO_2_-H_2_CO_3_ catalysis. These residues, like the catalytic domains, are highly conserved across species (Supplemental Fig. S10). In the CA4 dimer structure, the residues form a surface-exposed ridge that extends across almost 25 Å and is likely to form the binding site for SLAC1 (Figs. 1 and 2). Mutations of this surface—notably V^157A^, H^158A^, and K^160A^ substitutions—were sufficient to eliminate binding and suppress the CA-enhanced SLAC1 current without apparent impact on CA rescue of the catalytically-impaired *ΔNCE103* yeast (Fig. 2). Thus, the functions of CA4 in SLAC1 binding and in catalytic activity are structurally separable. Even so, overlaps between CA catalytic activity and channel binding suggest that SLAC1 binding is linked to CO_2_-H_2_CO_3_ catalysis, as is to be expected for CA4 to sense and regulate channel activity with pCO_2_.

### The mechanics of SLAC1 regulation by CA4

At present, the domain on SLAC1 that binds CA4 has yet to be resolved. Nonetheless, the motif for SLAC1 binding on CA4 offers some clues. Notably, both the membrane-associated CA4.1 and the cytosolic CA4.2 interacted with SLAC1 in mbSUS assays (Fig. 1). The two isoforms are sequence-identical apart from the absence in CA4.2 of the amphipathic N-terminus. Furthermore, the presence of charged residues associated with the V^157^HxK-centered binding motif on CA4 should focus efforts to screening for complementarily charged sites exposed on the cytosolic face of SLAC1.

**Fig. 6:**
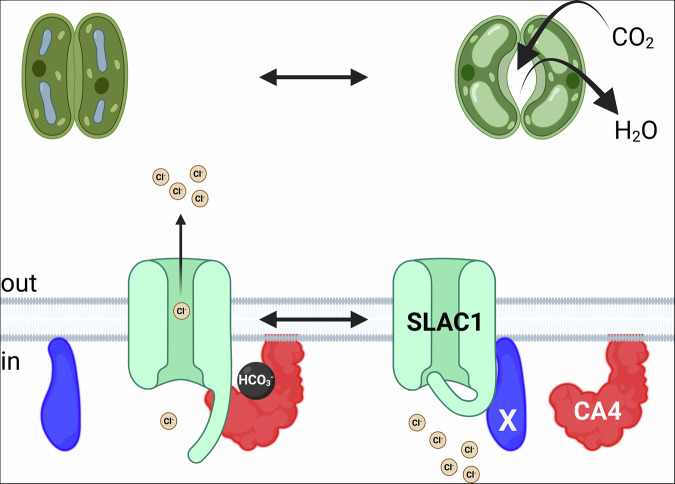
A mechanism coupling HCO_3_^-^ and CA4 binding to SLAC1 activation. Binding of HCO_3_^-^ to CA4 favours SLAC1-CA4 interaction, promotes SLAC1 activity to accelerate stomatal kinetics (Fig. 3). However, increasing CO_2_, and hence HCO_3_^-^, does not further enhance the current when SLAC1 and CA4 are expressed heterologously, whereas it does in vivo. One explanation is that a third component (X) is present in vivo - but not in the heterologous system - and normally must be displaced by CA4 when bound with HCO_3_^-^ to promote SLAC1 activity. Enhancing SLAC1 current favours membrane depolarisation, promotes Cl^-^ and K^+^ efflux, and thereby accelerates stomatal closure. Created in BioRender. Karnik, R. (2026) https://BioRender.com/0u75aph.

How might CA4 binding regulate SLAC1? Recent structure/function studies of SLAC1 have highlighted the cytosolic N- and C-termini of the anion channel that fold back on the internal surface of the protein and interact near the internal mouth of the channel pore to block anion passage^24^ (see Fig. 6). All evidence suggests that phosphorylation of two Ser residues, S^59^ and S^86^ weakens these interactions, unfolding the N- and C-termini and allowing anions to pass through the pore. One plausible interpretation, therefore, is that CA4 engages with these termini, possibly with Ser residues when phosphorylated and thereby negatively charged. Alternatively, CA4 may compete with the N- and C-termini for binding with sites at the base of the SLAC1 protein, possibly with the short cytosolic α-helix present between the second and third transmembrane domains of SLAC1^24,25^.

We suspect that an additional component is needed for dynamic regulation by CO_2_ of the anion channel. A relative enhancement of SLAC1 current similar to that in guard cells on raising CO_2_ from 0 to 1000 μbar was evident in oocytes co-expressing SLAC1 with CA4.1 ^wt^, but this enhancement was independent of CO_2_. By contrast, a CO_2_-dependence to the relative enhancement in SLAC1 was evident on CA4.1 expression in the plant (Fig. 4). It follows that an additional factor, present in the plant, is needed for a graded reponse to CO_2_/HCO_3_^-^. Given the positive impact on the current in oocytes, it is plausible that CA4 competes with a third partner for binding with SLAC1 (Fig. 6). The same reasoning may explain why previous expression studies failed to recover any CO_2_/HCO_3_^-^ sensitivity except when HCO_3_^-^ was raised to concentrations equivalent to over 100 times atmospheric CO_2_^28,29,47^. Apart from the dynamics with CO_2_, the effect clearly is to facilitate or stabilise the channel protein to favour channel activity at near-ambient pCO_2_ and HCO_3_^-^.

Best estimates indicate apparent K_1/2_ values for binding and SLAC1 activation of 650–700 μbar CO_2_ and, when equilibrated in solution, near 0.35 mM HCO_3_^-^ (Figs. 3 and 4). These values accord also with the normal physiological range of pCO_2_ values in the leaf that promote stomatal closing^16,46^ and they are supported by the known actions of CA4 in CO_2_ and HCO_3_^-^ binding. By contrast, reports of SLAC1 activation that is sensitive to mutation of residue R^256^ of the channel^47^ have required 11.5 mM HCO_3_^-^, a concentration that would be expected only on equilibration with an atmosphere of almost 4% CO_2_ or roughly 100 times ambient levels in the air. Indeed, the argument for direct binding of HCO_3_^-^ to SLAC1 is based solely in silico modelling; experimental evidence of binding that would validate the claim for sensing CO_2_/HCO_3_^-^ is lacking.

We stress that SLAC1 binding and regulation by CA4 does not discount recent evidence for HCO_3_^-^-mediated changes in phosphorylation and interaction of the HT1 kinase with MPK12^26^, but may be seen to complement these findings. Much as is the case for ion transport, multiple levels of regulation are a feature common to the responses of plants to environmental stimuli^58–61^. Nonetheless, HCO_3_^-^ is reported to promote HT1-dependent phosphorylation with a K_1/2_ around 7 mM^26^. For guard cells with a buffer capacity of 70 mM H^+^/pH unit and resting cytosolic pH near 7.5^59,62^, 7 mM HCO_3_^-^ is expected on equilibration with 30,000 μbar CO_2_, almost 100 times atmospheric pCO_2_ and far above the physiological range likely to occur within the leaf. Such a low apparent affinity must raise questions about the functional impact of HCO_3_^-^-mediated phosphorylation and whether the HCO_3_^-^ dependence of HT1 serves as a failsafe rather than a primary mechanism in CO_2_-evoked signalling.

### Implications for water use and assimilation

The immediate consequence of impaired CA4 binding in the plant is to eliminate SLAC1 current enhancement on moderately elevating pCO_2_. So, it is no surprise that the non-binding CA mutants slowed stomatal closure when compared to the CA4^wt^-complemented *ca1ca4* mutant plants (Fig. 4). The observations clearly show that Cl^-^ efflux becomes a rate-limiting factor in net solute and turgor loss for closure when the SLAC1 current is not elevated. As predicted by quantitative modelling (Supplemental Figs. 5, 6), increasing the apparent K_1/2_ for HCO_3_^-^ action on SLAC1, so decreasing its sensitivity to pC_i_, reduces the bias for membrane depolarisation that is needed to engage the R-type channels and accelerate solute efflux^7,8^. Reducing or eliminating the enhancement in SLAC1 current also slows stomatal opening through metabolic links that affect the capacity for K^+^ uptake, as was predicted and experimentally demonstrated previously^18^. Thus separate from its catalytic activty, the binding of CA4 with SLAC1 is a key factor defining channel activity and is central to enhancing stomatal kinetics, WUE and carbon assimilation (Figs. 4,5, Supplemental Figs. 5, 6).

Our findings have strategic implications for crop improvement. The SLAC1 channel and its homologues are a common feature of the stomatal guard cells^25,59,63–65^ and, for the few species that have been examined, are known to contribute to solute efflux for stomatal movements^63–65^. The genomes of these species also include a subset of CAs, alignment of which shows that all harbour the same VHxK-centered motif for SLAC1 binding (Supplemental Fig. 10). These findings highlight potential molecular targets that might be leveraged for gains in both WUE and photosynthetic carbon assimilation through engineering the corresponding binding sites in the guard cells of crop plants. CA binding to SLAC1 and its homologues may also prove important for understanding the altered CO_2_ sensitivities of guard cells in plants that carry out C_4_ photosynthesis^66–68^, as has been suggested for the K^+^ channels of these plants^69^.

Much effort in research to date has focused on the benefits of reducing stomatal densities, which decreases transpiration and improves WUE but can also reduce the diffusion of CO_2_ to the mesophyll and slow plant growth^2,70,71^. Similarly, altering ion pump and channel populations will affect stomatal conductance and photosynthesis, but generally at the expense either of WUE or of carbon assimilation^18,51,65,72,73^. These findings are underlined by mechanistic analyses of stomata^49,66,72^ that suggest strategies altering transporter populations alone are less effective in improving stomatal performance; instead, targeting the mechanics of transport control, including the regulation of ion channels, is the most promising approach.

Previous work demonstrated the efficacy of introducing a new conductance for K^+^, either with the addition of a synthetic K^+^ channel^52^ or through alterations to the gating of a K^+^ channel native to the guard cell^49^. Our discovery of CA4 binding in activating a major Cl^-^ channel opens the door to parallel manipulations of anion flux to further enhance guard cell membrane transport and accelerate stomatal movements. In short, the findings present a fresh avenue to ‘tuning’ native ion channel functionality in order to improve WUE and carbon assimilation while circumventing the often conflicting demands in conserving water while ensuring photosynthetic assimilation for growth.

## Methods

### Molecular biology, split-ubiquitin assays and biochemistry

Gateway™ entry clones for *SLAC1*, *CA1*, *CA3*, *CA4.1* and *CA4.2* coding sequencing were PCR amplified from complementary DNA and verified by restriction digestion and sequencing in pDONR207, pDONR221-P1P4 and pDONR221-P3P2 vectors. Primers used in this study are listed in Supplemental Data 1. Site-directed mutagenesis of *CA4.1* was carried out by PCR^74^ using Phusion® High-Fidelity DNA Polymerase (NEB, Hitchin, UK) and verified by sequencing.

For transformation, destination clones were generated using Multisite Three-fragment Gateway™ reactions. The 5’ element of the 3-kb fragment upstream of the native *CA4.1* promoter^35^ was amplified from genomic DNA of Col0 Arabidopsis and the products then cloned into entry clone using pENTR 5’-TOPO TA Cloning™ Kit (Thermofisher, Inchinnan, UK). Genes of interest were cloned used in multisite Gateway™ reactions. Destination clones were generated in pNX35-Dest and pMetYC-Dest for yeast mbSUS assays^33^, in pAG423GPD-Dest for carbonic anhydrase activity assays^42^, in pFRET-2in1-CC-Dest for subcellar localization and FRET/FLIM assays^44,75^, in pH7m34GW-Dest for stable transformations^76^, and in pGT-Dest for oocyte expression^33,77^. The kinase CIPK23 and its partner protein CBL1 were in the pGEMHE oocyte expression vector^33^ and were included in all oocyte transformations.

For co-immunoprecipitation analysis, total microsomal fractions were isolated as described previously^78^ using freshly-harvested Arabidopsis leaf tissue. Microsomal pellets were collected by centrifuging supernatants at 100,000 *g* at 4 °C for 1 h and resuspended in 5 mM phosphate buffer, pH 7.8, 330 mM sucrose, 0.1 mM EGTA, 0.1 mM EDTA-KOH, 100 mM phenylmethylsulfonyl fluoride, 1 mM DTT, and protease inhibitor cocktail. Samples were diluted with the binding buffer (PBS, 0.01% v/v Triton X-100, 0.05 mM DTT, 0.01% CHAPS, protease inhibitor) before incubating with GFP-Trap agarose beads (Chromotek, Munich, Germany) for 1 h at 4 °C with gentle shaking. Beads were collected and washed 3 times with binding buffer, with additional 150 mM NaCl, and bound proteins were eluted with 50 mM Tris–HCl, pH 6.8, 5% w/v SDS, 2 mM EDTA, 0.1% v/v Triton X-100, 50 mM DTT, 12% v/v glycerol, and 0.05% w/v bromophenol blue for subsequent analysis.

### Yeast growth assays

Protein-protein interactions were carried out by mating-based split-ubiquitin (mbSUS) assay using full-length SLAC1 in pMetYC-Dest and CA constructs in pNX35-Dest vectors to transform yeast THY.AP4 and THY.AP5 mating lines, respectively^32,33^. Transformed yeast colonies were selected for growth on complete synthetic media (CSM) minus leucine and methionine (CSM_-LM_) for THY.AP4, and without tryptophan, uracil and methionine (CSM_-TUM_) for THY.AP5. Mated yeast were selected on CSM minus leucine, tryptophan, uracil and methionine (CSM_-LTUM_), diluted in sterile water and dropped at 1.0, 0.1, 0.01 OD_600_ on CSM minus adenine, histidine, leucine, tryptophan, uracil and methionine (CSM_-AHLTUM_) as a positive test for bait-prey interactions. Growth on CSM_-AHLTUM_ with additions of 0, 5, 50 and 500 μM methionine was included to suppress bait expression as a test for specificity and strength of interactions. Growth on CSM_-LTUM_ was monitored for 24 h to confirm mating and on CSM_-AHLTUM_ for 72 h to test for protein interactions. The presence of yeast led to a steady-state of 600–800 μbar CO_2_ over both media after 24 h. Expression of bait and preys were verified by immunoblot using antibodies against VP16 and HA, respectively.

### Carbonic anhydrase activity

Wild-type and mutant CA4.1 in pAG423GPD-Dest were transformed in the yeast strain *ΔNCE103* lacking CA activity^42^. Transformed yeast colonies selected on CSM media lacking histidine and threonine (CSM_-HT_) under 5% CO_2_ at 30 °C. Pools of 10–15 colonies were resuspended in distilled water and dropped at 1.0, 0.1, and 0.01 OD_600_ for growth under 5% and ambient (0.4%) CO_2_ on CSM_-HT_. Growth was monitored for 72 h and protein expression verified by immunoblot.

### Recombinant proteins, custom antibodies and immunochemistry

Recombinant peptide synthesis and gel filtration assays for N-terminal multimers were carried out as before^79^. Gel filtration eluates were assessed against molecular weight standards and band intensities quantified against known molar quantities of the same proteins.

Polyclonal antibodies were raised in rabbits by immunization with unique synthetic peptide NKIKEEHKDLSYDDQ for CA4 (Eurogentec, Seraing, Belgium) and with the N-terminal 189 residues of SLAC1 for the channel (Agrisera, Vännäs, Sweden). Antibody specificity was verified by reaction with the synthetic antigens used for immunization and by loss of protein-specific bands in immunoblots probed with antigen-saturated antibodies (see Fig. 1 and Supplemental Fig. 2).

Immunoblots were carried out on nitrocellulose membranes and separated proteins probed with commercial antibodies αVP16 (Abcam, Cambridge, UK, ab4808, 1:10,000), αHA (Abcam, ab137838,1:10,000), αGFP (Abcam, ab6556, 1:5000), αmCherry (Abcam, ab167453, 1:5000), and αBIP (Agrisera, AS09481, 1:10,000), and with αCA4 (this study, 1:10,000 dilution) and αSLAC1 (this study, 1:1000 dilution). Secondary goat-αrabbit as the horseradish-peroxidase conjugate (Abcam, ab6721, 1: 20,000) was used and cross-reacting bands were visualized using West Femto Super Signal chemiluminescence detection (Thermofisher, Inchinnan, UK) imaged on a Fusion Chemiluminescence imager (Vilber, France). Total proteins were visualized by staining the membrane with Coomassie. Band density was measured by densitometry using the ImageJ software.

### Plant growth, transformation and whole-plant physiology

Tobacco (*Nicotiana tabacum*) were grown and leaves transformed by *Agrobacterium* infiltration^80^. Arabidopsis (*Arabidopsis thaliana*) wild-type Col0, and the *ca1ca4* double mutant^29^ were from the Nottingham Arabidopsis Stock Centre.

For co-IP analysis, *ca1ca4* mutant Arabidopsis seedlings were grown and transformed by co-cultivation with *Agrobacterium tumefaciens* GV3101 harbouring the genes of interest in the 2in1 vectors pFRETgc-2in1-CC-Dest as described previously^44,81^.

Stable transgenic lines were generated in the *ca1ca4* mutant by floral dipping^82^ and the T1 generation selected following growth on 25 mg/L hygromycin. At least two independent lines per construct were carried forward to the T2 generation and were also used for analysis. Seed were sterilized and grown at 22:18 ^o^C and 9:15 h light:dark cycles at 60% relative humidity.

Gas exchange measurements using LICOR 6800 gas exchange systems (Lincoln, USA) and growth experiments were carried out as described previously^18,19,51^. Soil water content was monitored using a ML3 moisture sensor (DeltaT Devices, Cambridge UK) and plants watered daily to maintain 10 ± 5% soil water content.

Plant growth assays were carried out under 200, 400 and 1000 μbar CO_2_ in GEN1000 growth chambers (Conviron, Isleham, UK) with a total daily fluence of 4.5 mol m^−2^ PAR. For comparisons between constant and fluctuating light regimes, plants were grown either under constant light of 140 μmol m^−2^ s^−1^ or under light that stepped at intervals between fluence rates from 40 to 240 μmol m^−2^ s^−1^. Rosette areas were acquired using ImageJ v.1.54i after 5 wk and fresh and dry weights determined for each plant.

Total Cl^-^ and NO_3_^-^ contents were measured from plants grown from sterilised seed, selected and grown on defined liquid media^83^ with 10 mM Cl^-^ and 1 mM NO_3_^-^. Plants were harvested after 21 d, rinsed and surface dried against filter paper before extraction. Total Cl^-^ and NO_3_^-^ were quantified on a fresh-weight basis by standardized, colorimetric assays, for Cl^-^ using Hg(SCN)_2_ reduction with Fe^3+^ to form Fe(SCN)_3_^84^ and for NO_3_^-^ using acidic nitration of salicylic acid^85^.

### Confocal microscopy

Transformed tobacco and Arabidopsis leaves were imaged on a Leica SP8 SMD confocal microscope equipped with 20x/0.85 NA dry and 40x/1.3 NA oil lenses and hybrid GaAs detectors (Leica, Wetzlar, Germany). Fluorescence was excited with the 488 and 552 nm laser lines for GFP and mCherry fluorescence and fluorescence collected across 495–550 and 580–630 nm, respectively. Chloroplast autofluorescence was excited with 488 nm light and collected across 630–690 nm. Laser intensities and detector gains were standardized between sets of experiments for quantitative analysis. Fluorescence signals were analysed using ImageJ v.1.54i^86^.

Fluorescence lifetimes of GFP without and with the mCherry acceptor were measured by time-correlated single-photon-counting using a PicoHarp 300 module and were analysed using FLIMfit software^87^. GFP was excited with a 470 nm laser line at 40 MHz and the emission recorded at 496–525 nm to ensure counts of at least 1000 photons per pixel^88^. The instrument response function, corresponding to the laser pulse convoluted with the detection response, was determined at the emission wavelength by measuring the fluorescence decay of Erythrosine B in KI solution that shows fast recovery and decay^89,90^. Lifetimes were determined by reconvolution of the data and fitting of the decay to a sum of two exponentials. To avoid signal contamination and potential miscalculations, only regions of interest (ROI) covering the plasma membrane area were used, and only fittings yielding chi-squared values of 0.9–1.4 were considered. At least 8 independent experiments with at least 10 cells per experiment were analysed for each construct.

### Electrophysiology

The activity of SLAC1 expressed in *Xenopus* oocytes (Ecocyte Bioscience, Dortmund, Germany) was measured using standard protocols and a two-electrode voltage clamp^77,91,92^. cRNAs generated after linearising cDNAs were transcribed with HiScribe T7 ARCA mRNA kit (New England Biosciences, Poole) and injected at 1 ng/oocyte. cRNAs were co-injected after mixing for molar ratios of 2:1 SLAC1:CIPK23 and 1:1 CBL1:SLAC1. CA4.1 ^wt^ and its mutants were included at molar ratios to SLAC1, as indicated. Recordings were carried out 48–72 h after injection and oocytes were collected and analyzed for protein expression. Oocytes were superfused with modified ND96 buffer comprising 100 mM KCl, 1.5 mM MgCl_2_, 1 mM CaCl_2_ and 10 mM HEPES-NaOH, pH 7.4 adjusted for osmotic balance^77,93^. All recordings were carried out using Henry’s EP suite (v.3.5.5.5, Y-Science, Glasgow, UK).

Currents from intact guard cells in epidermal peels were recorded using double-barrelled microelectrodes and Henry’s EP suite (Y-Science, Glasgow). Electrodes were filled with 400 mM CsCl and guard cells were superfused with 15 mM CsCl and 15 mM Tetraethylammonium chloride (TEA-Cl) in 5 mM Ca^2+^-MES, pH 6.1 ([Ca^2+^] = 1 mM) to eliminate the background of K^+^ channel currents^18,51^. Voltage was clamped in cycles from a holding voltage of +30 mV to activate the SLAC1 current with negative-going steps between +30 and −180 mV to resolve the current. Currents were analyzed using Henry’s EP suite and SigmaPlot 11.2 (Systat Software, Inc., USA) as described previously^19,21,48,92,94^.

### OnGuard modelling and statistics

Quantitative modelling using the OnGuard3e platform was carried out as described previously^19,51,95–97^. Model parameters are listed in Supplementary Data 2. As OnGuard outputs are determined by the interactions of the ordinary differential equations that describe each of the underlying processes, statistical analysis of these outputs is meaningless. All other results are reported as means ± SE of *n* independent experiments. Significance was determined by one-way Analysis of Variance (ANOVA), as appropriate with post-hoc analysis (Student-Newman-Keuls, Holm-Sidek and Tukey), and is indicated at *P* < 0.05 unless otherwise stated.

### Accession numbers

Sequence data from this work can be found in the Arabidopsis Genome Initiative and GenBank/EMBL databases under accession numbers AT1G12480.1 (SLAC1), AT5G37500.2 (GORK), AT3G01500.2 (CA1), AT1G23730.1 (CA3), AT1G70410.2 (CA4.1) and AT1G70410.1 (CA4.2).

### Reporting summary

Further information on research design is available in the Nature Portfolio Reporting Summary linked to this article.

## Supplementary information

Supplementary information

Description of Additional Supplementary Files

Supplementary Data 1

Supplementary Data 2

Reporting Summary

Transparent Peer Review file

## Source data

Source Data

## Data Availability

Data generated and analysed during this study are included in the article and its supplementary files. Unique biological materials are available on reasonable request to the corresponding author. Source data are provided with this paper.

## Code availability

The OnGuard3 platform and the model parameter sets are freely available to academic users and may be downloaded from www.plantscienceglasgow.org.
