# Supplementary material for: A guard cell carbonic anhydrase binds and regulates SLAC1 separate from its catalytic activity
Source: Nat Commun. 2026 Mar 13;17:3911. doi: 10.1038/s41467-026-70596-9 (PMC13128974; doi:10.1038/s41467-026-70596-9)
Supplement: Supplementary file 1 — Supplementary information [file 41467_2026_70596_MOESM1_ESM.pdf]

A guard cell carbonic anhydrase binds and regulates SLAC1 separate from its catalytic activity
Lingfeng Xia et al.

Supplemental Figures

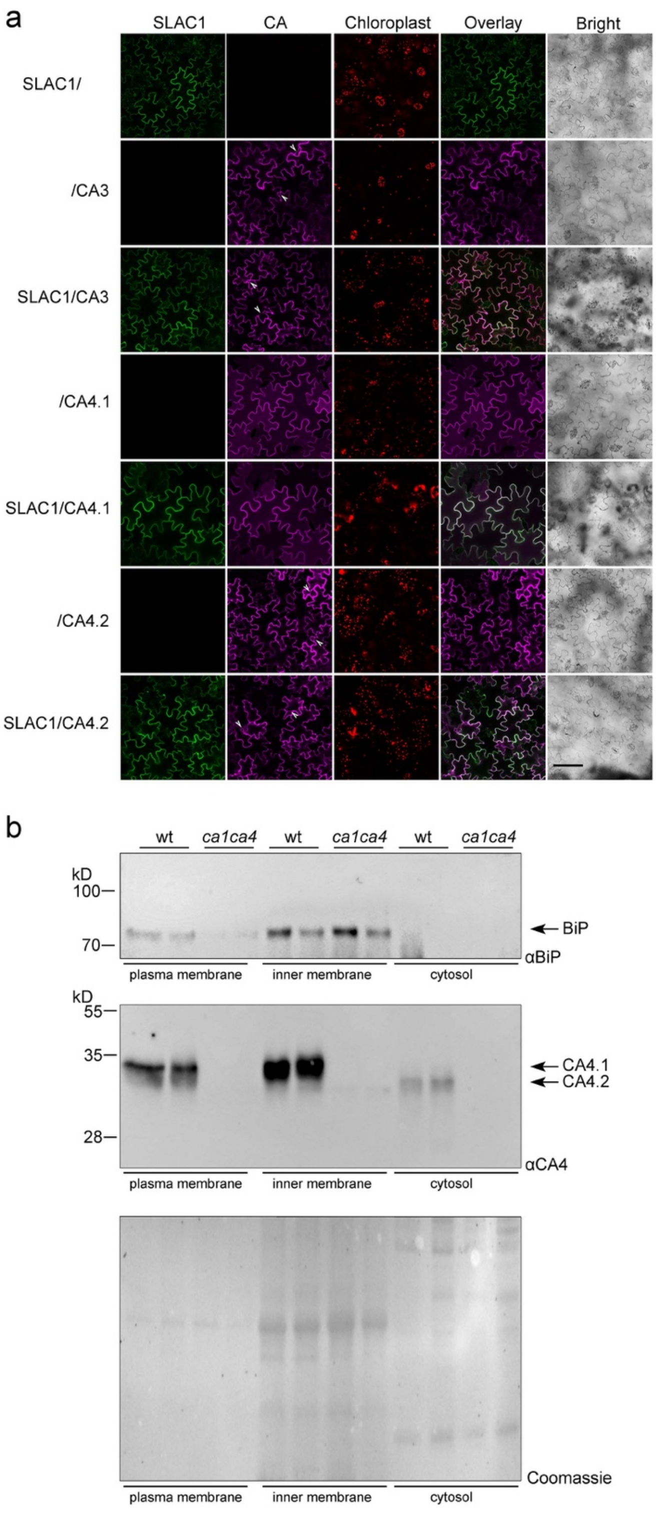

Supplemental Figure 1. **β-carbonic anhydrase isoforms CA4.1 and CA4.2 show distinct localisations**. (a) Representative confocal images of SLAC1-GFP and CA-mCherry fusions expressed alone and together in tobacco leaf epidermis show localisation of the channel and CA4.1 to the cell periphery. Corresponding chlorophyll fluorescence (Chloroplast), and brightfield (Bright) images are shown along with GFP and mCherry overlays (Overlay). The mCherry signal from β-carbonic anhydrases CA3 and CA4.2 are present in trans-vacuolar cytoplasmic strands (*arrowheads*) that are also evident on co-expression with SLAC1. Scale bar, 100 µm. (b) Immunoblot analysis of total Arabidopsis wild type and *ca1ca4* mutant leaf cell lysates separated into plasma membrane, inner membrane, and cytosol fractions, two preparations each were analysed. Endoplasmic reticulum resident protein, BiP detected using αBiP antibody suggests >85% purity of plasma membrane fractions (*top panel*). Native carbonic anhydrase isoforms CA4.1 (30.8 kDa) and, CA4.2 (28.4 kDa) were detected using custom synthesised αCA4 antibody in wild type Arabidopsis but not in the *ca1ca4* mutants, indicating antibody specificity. CA4.1 was detected in plasma membrane and inner membrane fractions, while CA4.2 was detected in cytosolic fractions (*middle panel*). Total protein in each lane was

detected using Coomassie staining of the immunoblot membrane (*bottom panel*). Note the lower levels of total protein present in the plasma membrane fraction.

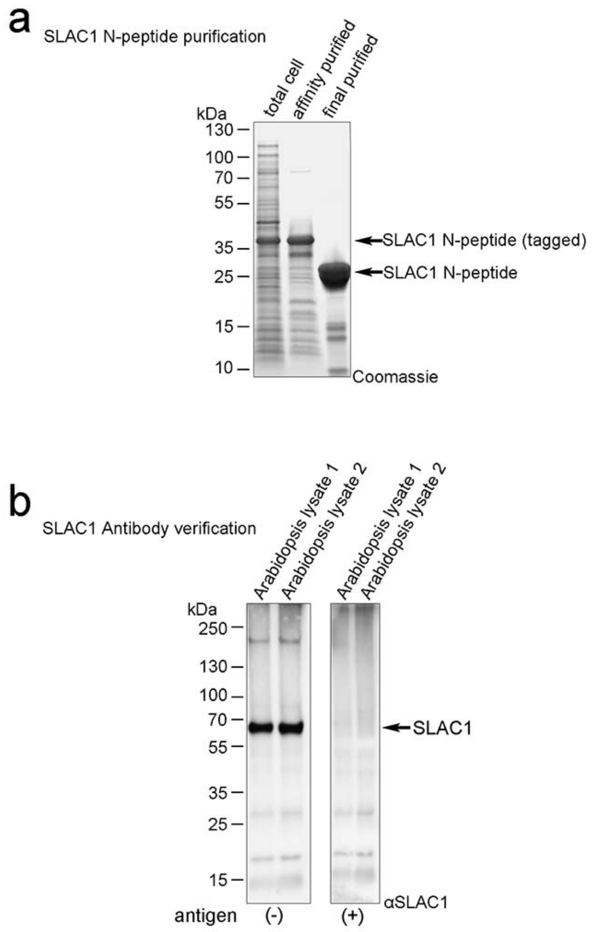

Supplemental Figure 2. **Purification of the SLAC1 antigen and verification of the SLAC1 polyclonal antibody.**
(a) Coomassie stained SDS-PAGE gel, detecting protein in E.coli cell lysates expressing (*left to right*) SLAC1 N-peptide (residues 1-189) with a 6xFLAG-StrepII tag, affinity purified 6xFLAG-StrepII-tagged SLAC1 N-peptide, and the final purified SLAC1 N-peptide peptide (21.6 kDa) following enzymatic cleavage. The purified peptide was isolated using size exclusion chromatography and was used as antigen for immunisation to generate the αSLAC1 polyclonal antibody.
(b) Immunoblots verifying the specificity of αSLAC1 polyclonal antibody. Total protein in wild-type Arabidopsis leaf lysates separated by SDS-PAGE and probed with anti-SLAC1 antibodies. The lysates were incubated without (-) or with (+) the purified SLAC1 N-peptide to quench αSLAC1 binding. Antibodies detected the native SLAC1 protein (63.3 kDa) band (*left*), but not when binding was quenched (right). The weak band around 190 kDa is consistent with SLAC1 trimers.

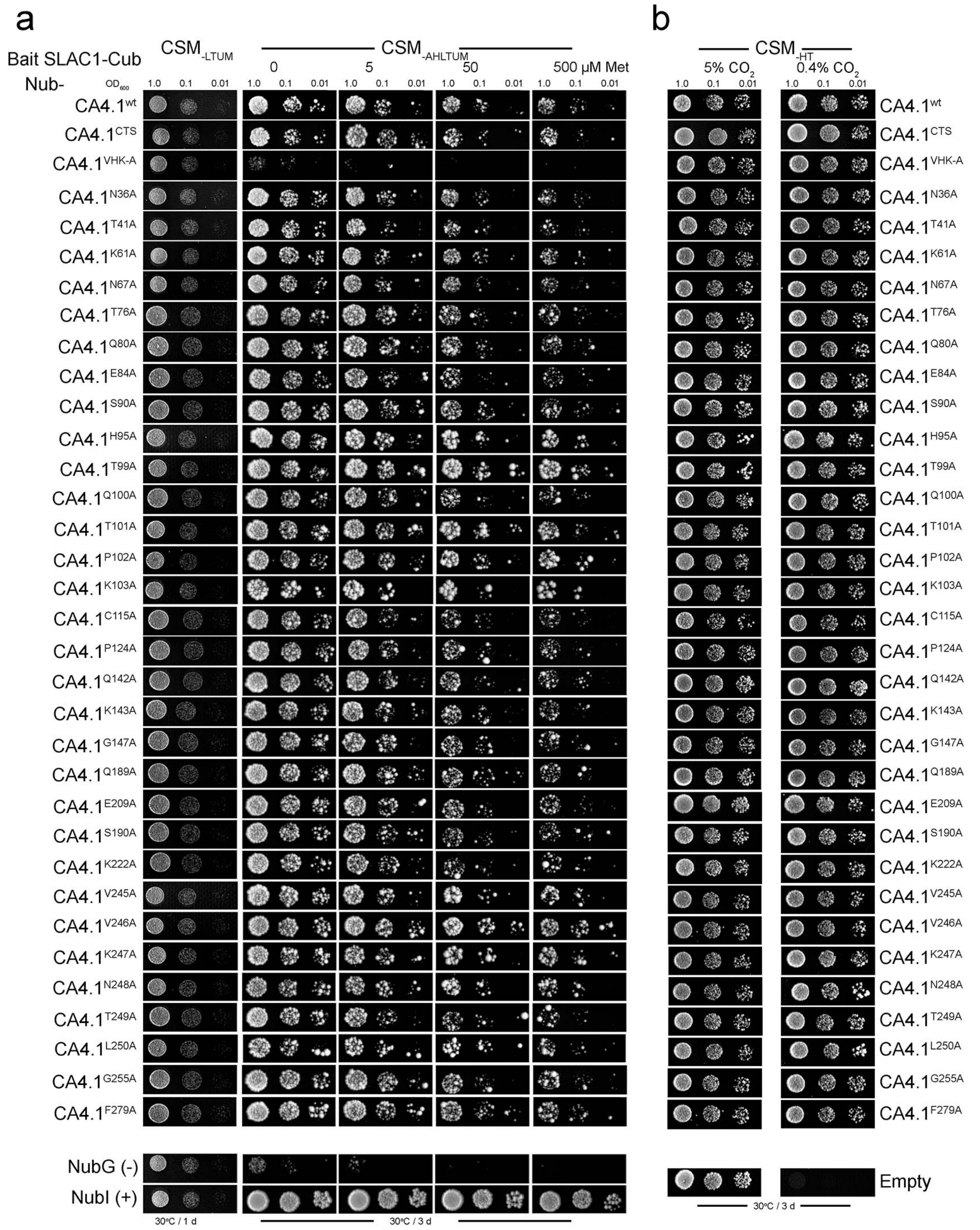

Supplemental Figure 3. **SLAC1 anion channel interaction with CA4.1 and CA catalytic activity are unaffected by Ala substitutions of a majority of residues predicted to reside at the protein surface.**

(a) Yeast mating-based split-ubiquitin (mbSUS) assay for binding with SLAC1-Cub as bait with Nub-fusions of β-carbonic anhydrase CA4.1 and the mutations indicated as preys. Controls ([-], NubG; [+], NubI) included for reference. One of five independent experiments, all yielding similar results. Yeast diploids dropped at 1.0, 0.1 and 0.01 OD_{600} spotted (*left to right*) on complete synthetic medium without Trp, Leu, Ura and Met (CSM_{-LTUM}) to verify mating, on CSM without Trp, Leu, Ura, Ade, His and Met ($CSM_{-LTUMAH}$) to verify adenine- and histidine-independent growth, and with Met

additions as indicated to suppress bait expression. Immunoblots are included in Supplemental Fig. 10.

(b) Complementation of the yeast *ΔNCE103* mutant that normally requires 5% CO_2 shows growth rescue at ambient CO_2 with the same mutations (a). The *ΔNCE103* mutant background (Empty) and complemented yeast plated on synthetic media minus histidine and threonine (CSM_{-HT}) to verify the background and grown under ambient (0.4%) and 5% CO_2. Immunoblots are included in Supplemental Fig. 10.

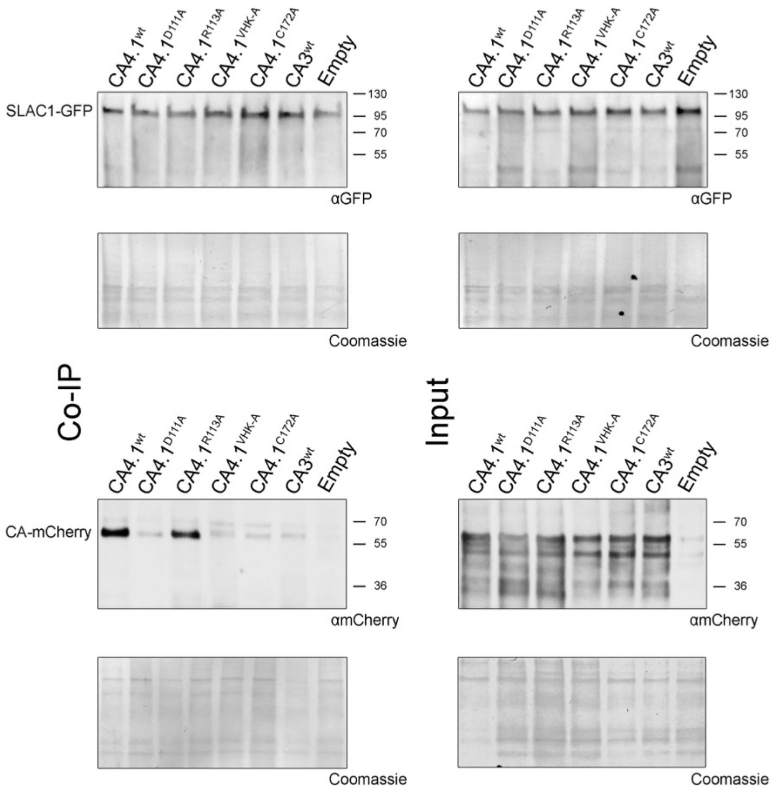

Supplemental Figure 4. **Verification of binding by co-immunoprecipitation with SLAC1 of CA3, CA4.1^{wt} and selected CA4.1 mutants.**

Co-immunoprecipitation (co-IP) of selected carbonic anhydrases, tagged with mCherry and expressed in *ca1ca4* mutant Arabidopsis together with SLAC1-GFP (see **Methods**). One of three independent experiments, each including one sample expressing SLAC1-GFP alone (Empty), all yielding similar results. SLAC1-bound proteins were recovered after incubating leaf fractions with GFP-Trap agarose beads and elution. Aliquots (10 μg protein) for input (*right*) and co-IP (*left*) were separated by SDS-PAGE, analysed by immunoblot using αGFP and αmCherry primary antibodies, and gel loading verified by Coomassie staining. With SLAC1-GFP, co-IP showed strong binding with mCherry-CA4.1^{wt} and the mCherry-CA4.1^{R113A} mutant. Little or no binding was evident when SLAC1-GFP was co-expressed with the mutants CA4.1^{D111A}, CA4.1^{VHK-A} and CA4.1^{C172A}, and with CA3 (*bottom left*).

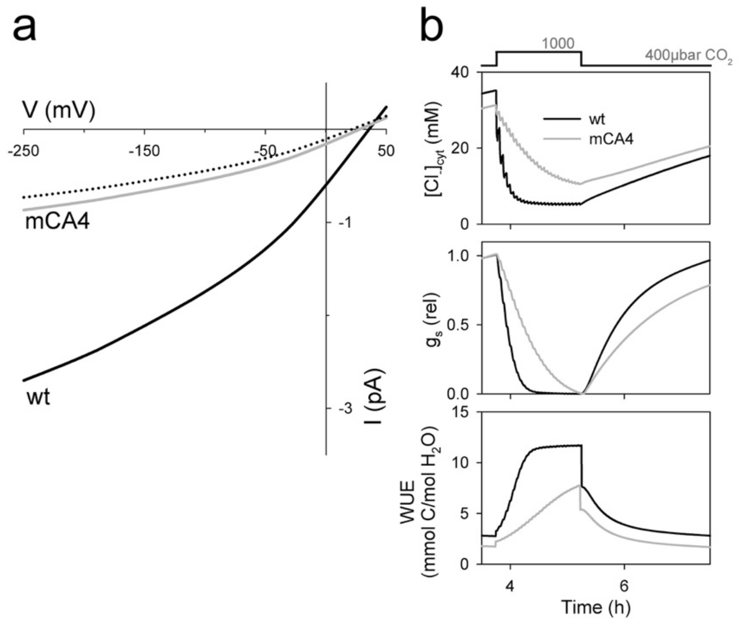

Supplemental Figure 5. **OnGuard3 modelling predicts impaired stomatal kinetics and water use efficiency (WUE) on reducing the CO_2 sensitivity of SLAC1.**
OnGuard3 modelling outputs for wild-type Arabidopsis (wt, black lines) and for a mutated CA4 (mCA4, grey lines) that increases the apparent K_d for SLAC1 activation by HCO_3^- from 0.3 to 3 mM.
(a) Model current-voltage (IV) curves following a step from 400 to 1000 μbar CO_2. The dotted line corresponds to the background SLAC1 current prior to the CO_2 step in each case.
(b) Modelled time courses for cytosolic [Cl$^-$] ([Cl$^-$]$_{cyt}$), relative stomatal conductance (g_s), and water use efficiency (WUE) with a 1.5 h step from 400 to 1000 μbar CO_2. Predicted halftimes for g_s with stomatal closing and opening are 7.1 and 28.0 min (wt), and 24.6 and 56.8 min (mCA4).

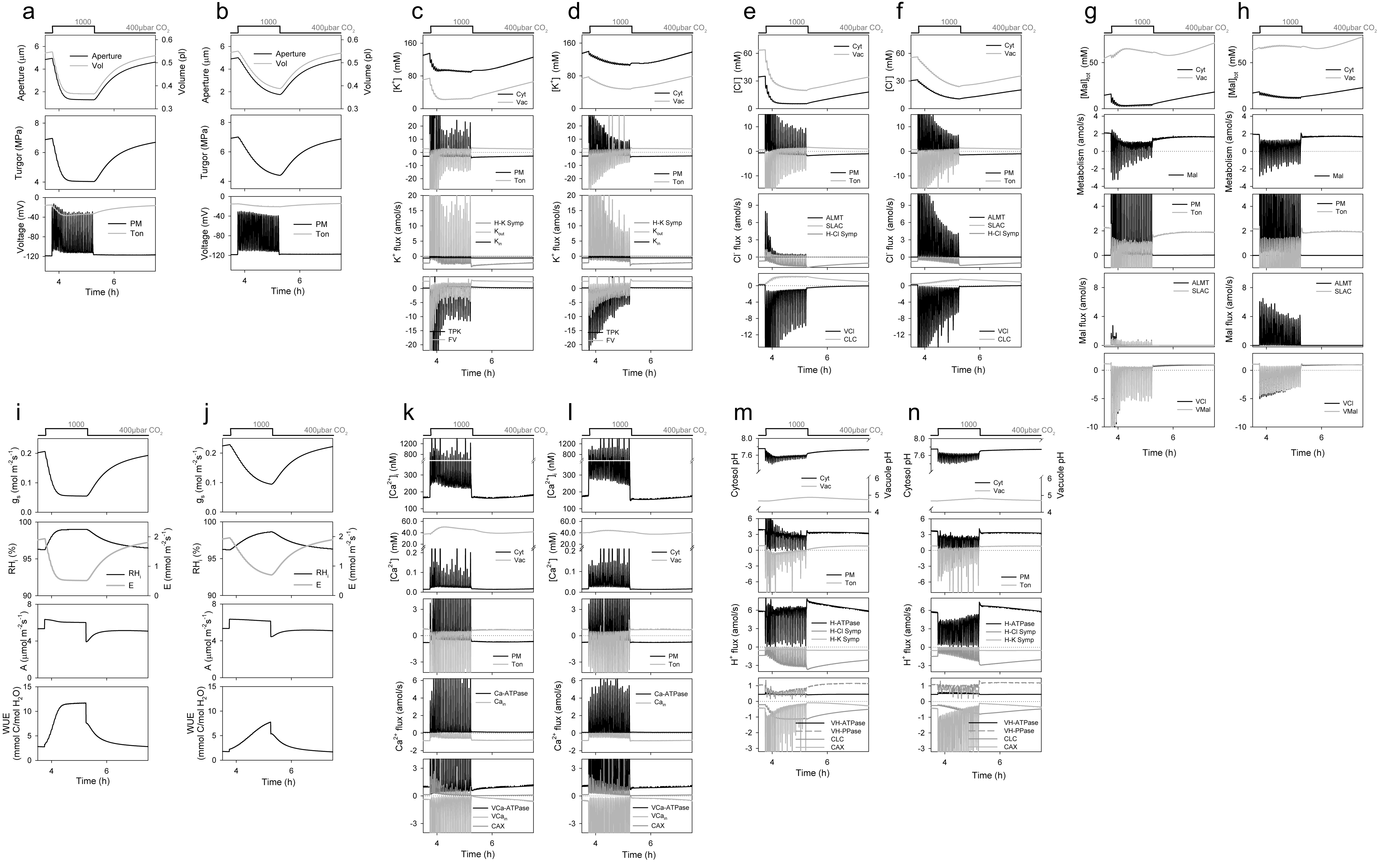
a
b
c
d
e
f
g
h
i
j
k
l
m
n
1000
400μbar CO2
Aperture (μm)
Volume (pl)
Aperture
Vol
Turgor (MPa)
Voltage (mV)
PM
Ton
Time (h)
[K+] (mM)
Cyt
Vac
K+ flux (amol/s)
H-K Symp
Kout
Kin
TPK
FV
[Cl-] (mM)
Cl- flux (amol/s)
ALMT
SLAC
H-Cl Symp
VCl
CLC
[Mal]tot (mM)
Metabolism (amol/s)
Mal
Mal flux (amol/s)
VMal
gs (mol m-2s-1)
RHi (%)
E (mmol m-2s-1)
RHi
E
A (μmol m-2s-1)
WUE (mmol C/mol H2O)
[Ca2+]i (nM)
[Ca2+] (mM)
Ca2+ flux (amol/s)
Ca-ATPase
Cain
VCa-ATPase
VCain
CAX
Cytosol pH
Vacuole pH
H+ flux (amol/s)
H-ATPase
VH-ATPase
VH-PPase

Supplemental Figure 6. **OnGuard3 model output comparison highlights the impaired Cl^- flux through the SLAC1 channel on reducing its CO_2 sensitivity**
Model outputs generated using a 1.5-h CO_2 step from 400 to 1000 µbar. Model parameters are listed in Appendix 1. Graphs are separated in column pairs for the wild-type (a,c,e,g,i,k and m) and with an increase in apparent K_d for HCO_3^- activation of SLAC1 (mCA4; b,d,f,h,j,l and n; see also Supplemental Fig. 4). Outputs are for aperture, cell volume, turgor and voltage (a,b); K^+ content and flux (c,d); Cl^- content and flux (e,f); Mal content, synthesis and flux (g,h); assimilation, transpiration, internal relative humidity (%RH), stomatal conductance, g_s, and water use efficiency (WUE) (i,j); Ca^{2+} free concentration, total content and flux (k,l); and pH and H^+ flux (m,n). Note that positive flux is defined as movement of the ionic species (not charge) out of the cytosol, either across the plasma membrane or the tonoplast. Mal synthesis is indicated by positive values (g,h). For each ion, the total flux across the plasma membrane (PM) and tonoplast (Ton) is given with the individual flux components for the two membranes shown in the two panels below. Abbreviations for the individual transporters cross-reference to the list in Appendix A. Oscillations in a number of fluxes are a consequence of the corresponding oscillations in voltage and cytosolic free $[Ca^{2+}]$ ($[Ca^{2+}]_i$) triggered with the step change in CO_2.

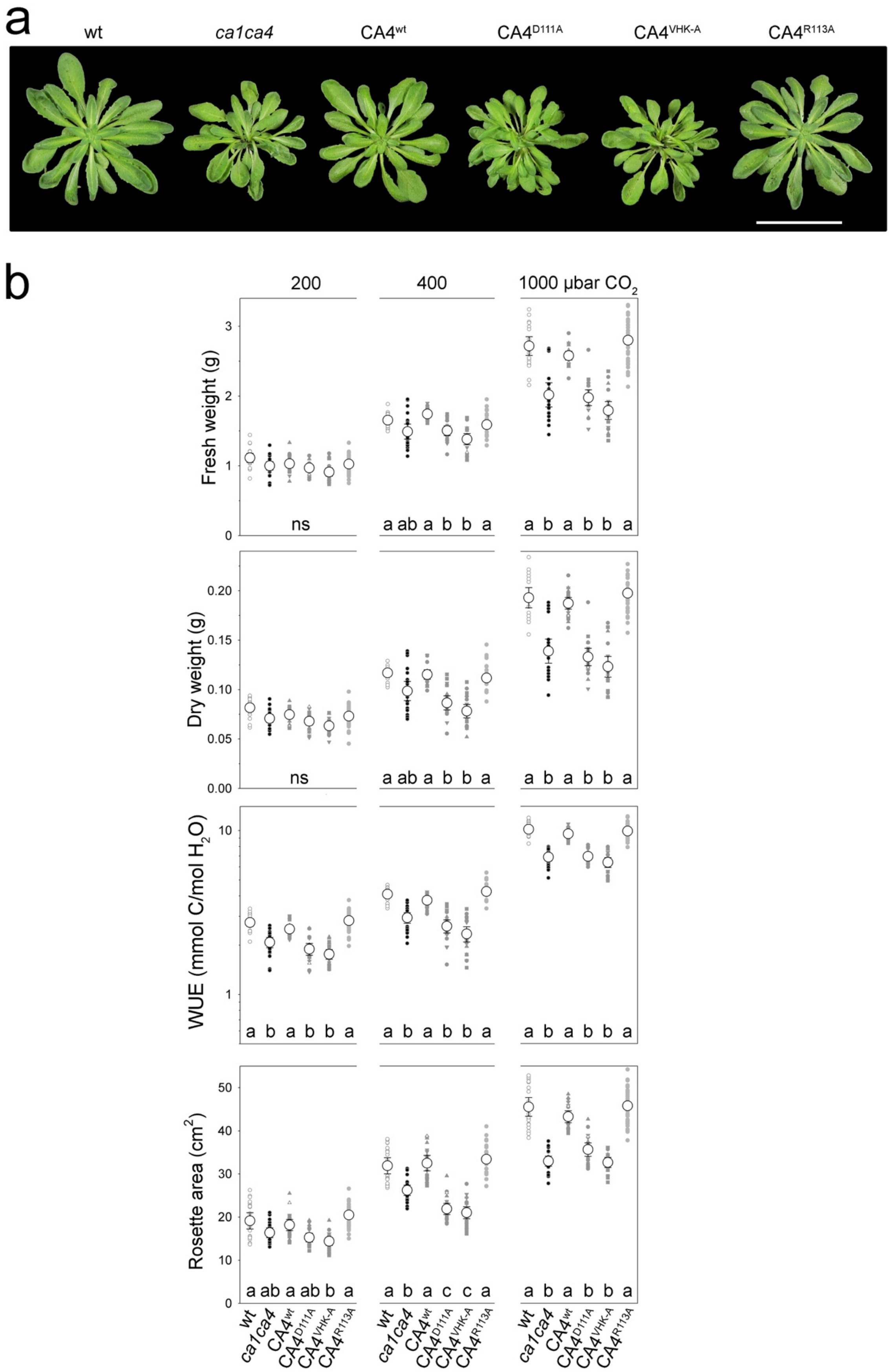
a
wt
ca1ca4
CA4wt
CA4D111A
CA4VHK-A
CA4R113A
b
200
400
1000 µbar CO2
Fresh weight (g)
Dry weight (g)
WUE (mmol C/mol H2O)
Rosette area (cm2)
ns
a ab a b b a
a b a b b a
ns
a ab a b b a
a b a b b a
a b a b b a
a b a b b a
a b a b b a
a ab a ab b a
a b a c c a
a b a b b a
wt
ca1ca4
CA4wt
CA4D111A
CA4VHK-A
CA4R113A

Supplemental Figure 7. **Impairing β-carbonic anhydrase CA4.1-SLAC1 binding leads to reduced growth and water use efficiency (WUE) in non-varying daylight**.
(a) Representative rosettes of wild-type Arabidopsis (wt), the *ca1ca4* mutant background, and plants of the *ca1ca4* background complemented with $CA4.1^{wt}$, $CA4.1^{VHK\text{-}A}$, $CA4.1^{D111A}$ and $CA4.1^{R113A}$ after 5 wk growth under constant daylight at 400 μbar CO_2. Scale: 5 cm.
(b) Analysis of plant growth under constant daylight with 200, 400 and 1000 μbar CO_2 as indicated. Data are (*top to bottom*) fresh weight, dry weight, WUE and rosette area. Data points for each genotype are independent plants and transformants (*small symbols*) and corresponding means ±SEM (*large open symbols*). Significant differences at $P<0.02$ within each CO_2 treatment are indicated by lettering and with ns to indicate not significant. Note that WUE values are plotted on a logarithmic scale.

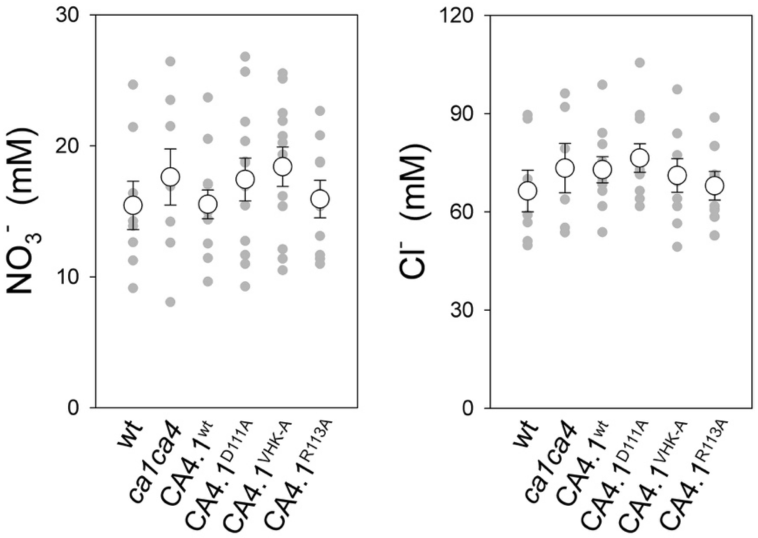

Supplemental Figure 8. **Total NO_3^- and Cl^- concentrations are not appreciably affected in the *ca1ca4* double mutant or its complementations.**

Total NO_3^- and Cl^- from wild-type Arabidopsis (wt), the *ca1ca4* mutant background, and plants of the *ca1ca4* background complemented with *CA4.1^{wt}*, *CA4.1^{D111A}* and *CA4.1^{VHK-A}* after 21 d growth on defined liquid media[83] with 10 mM Cl^- and 1 mM NO_3^- at 400 μbar CO_2 and non-varying daylight. Data are from independent transformants (small gray symbols) with means ±SEM (large open symbols) and are reported on a total volume (1:1 g:ml) basis. No significant differences at P<0.05 were evident between the different genotypes for either anion.

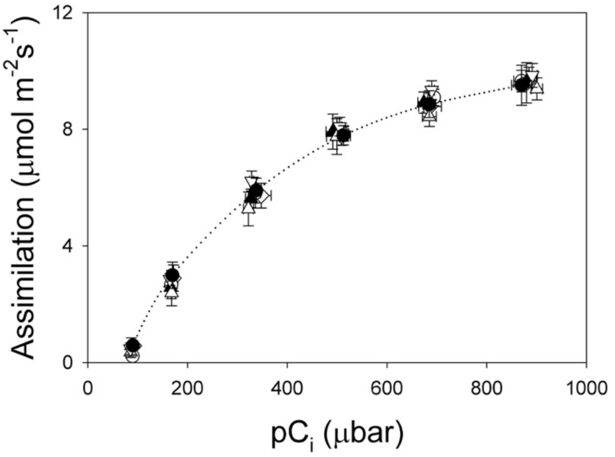

Supplemental Figure 9. **β-carbonic anhydrase CA4.1 mutants do not alter intrinsic photosynthetic capacity**

Assimilation rates as a function of the calculated CO_2 inside the leaf (pC_i) for wild-type Arabidopsis (●), the *ca1ca4* mutant (❍), and with CA4.1^{wt} (▲), CA4.1^{VHK-A} (△), CA4.1^{D111A} (▽), and CA4.1^{R113A} (■) under saturating photosynthetically-active radiation (PAR, 600 μmol m^{-2} s^{-1}). Data are means ±SE of n=5 independent

experiments for each genotype. CO_2 assimilation rates of the mutants were statistically indistinguishable from wild-type plants across the physiological range of internal CO_2 concentrations. Data for the wild-type plants are shown fitted to an empirical, second-order polynomial (dotted line) as a visual guide.

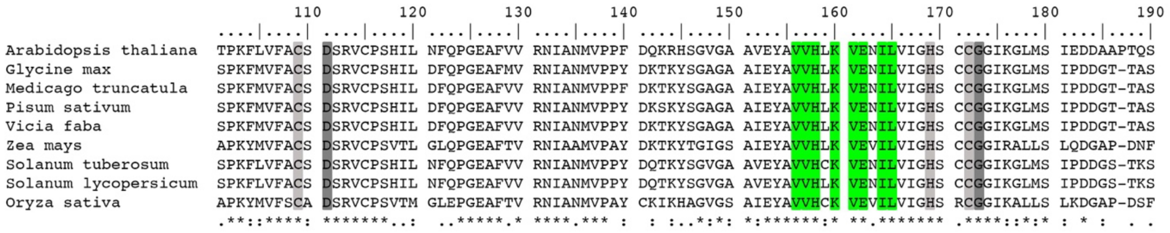

Supplemental Figure 10. **Amino acid alignment of the central domain of Arabidopsis CA4.1 with orthologous β-carbonic anhydrases of selected mono- and dicotyledonous species**.

Gene (NCBI) identifiers are for *Arabidopsis thaliana* (NP_849872.1), *Glycine max* (XP_006574621.1), *Medicago truncatula* (XP_024640294.1), *Pisum sativum* (XP_050905492.1), *Vicia faba* (KAL5080040.1), *Zea mays* (XP_008655227.1), *Solanum tuberosum* (XP_006365235.1), *Solanum lycopersicum* (NP_001296992.1), and *Oryza sativa* (XP_066167856.1). Residues in grey are those binding the CA substrates and zinc cofactor (see also Fig. 1); residues in green are those identified as the SLAC1-binding motif. Residue numbering is for Arabidopsis CA4.1.

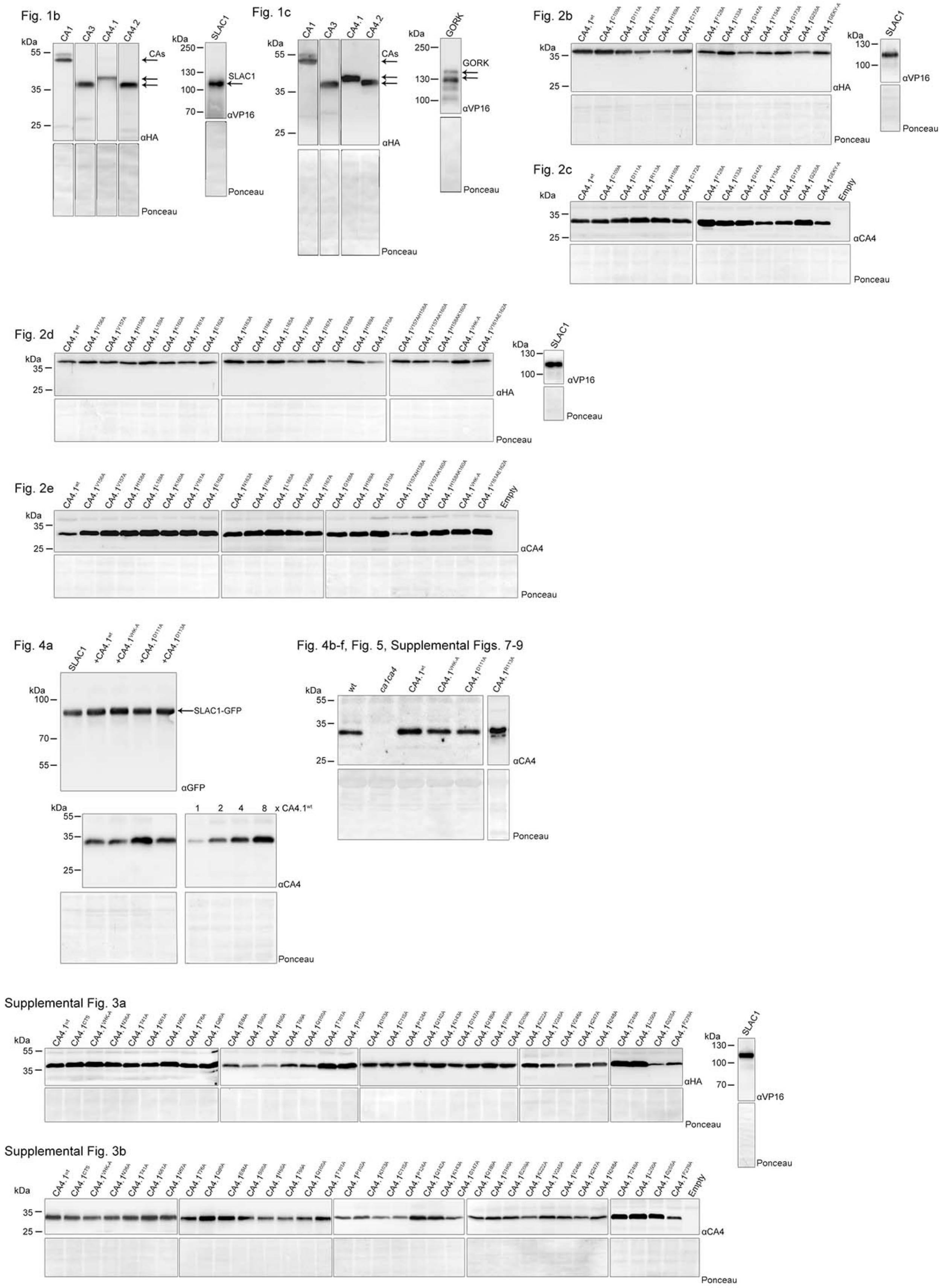

Supplemental Figure 11. **Immunoblot analysis for mbSUS assays, oocyte electrophysiology and stable Arabidopsis transformants**

Immunoblots for the data shown in the figures as indicated. Gels were loaded with 20 µg total protein/lane following extraction and probed as described (see Methods). Note the higher molecular weights expected for 2PA-PLV fusions of mbSUS baits.[47]
