# Supplementary material for: A guard cell carbonic anhydrase binds and regulates SLAC1 separate from its catalytic activity
Source: Nat Commun. 2026 Mar 13;17:3911. doi: 10.1038/s41467-026-70596-9 (PMC13128974; doi:10.1038/s41467-026-70596-9)
Supplement: Supplementary file 2 — Description of Additional Supplementary Files [file 41467_2026_70596_MOESM2_ESM.pdf]

Description of Additional Supplementary Files

File Name: Supplementary Data 1

Description: Primers and entry clones - Entry clones.

File Name: Supplementary Data 2

Description: OnGuard3 model parameters.
