# Supplementary material for: A guard cell carbonic anhydrase binds and regulates SLAC1 separate from its catalytic activity
Source: Nat Commun. 2026 Mar 13;17:3911. doi: 10.1038/s41467-026-70596-9 (PMC13128974; doi:10.1038/s41467-026-70596-9)
Supplement: Supplementary file 3 — Supplementary Data 1 [file 41467_2026_70596_MOESM3_ESM.pdf]

Supplmeentary Data 1. Primers and entry clones - Entry clones

Entry clones		Primer
1	pDONR207-L1-bCA4.1-ST-L2	1;3
2	pDONR207-L1-bCA4.2-ST-L2	2;3
3	pDONR207-L1-bCA1-ST-L2	5;6
4	pDONR207-L1-bCA3-ST-L2	7;8
5	pDONR207-L1-SLAC1-WO-L2	10;11
6	pDONR221-L3-SLAC1-WO-L2	12;11
7	pDONR221-L1-bCA4.1-WO-L4	1;4
8	pDONR221-L1-bCA4.2-WO-L4	2;4
9	pDONR221-L1-bCA3-WO-L4	7;9
10	pDONR221-L1-bCA4.1-Q100A_T101A_P102A_K103A-W	1;4
11	pDONR221-L1-bCA4.1-V161A_E162A-WO-L4	1;4
12	pDONR221-L1-bCA4.1-K247A_N248A_T249A_L250A-W	1;4
13	pDONR221-L1-bCA4.1-V157A_H158A_K160A-WO-L4	1;4
14	pDONR221-L1-bCA4.1-T83A_E84A_K85A_Y86A-WO-L4	1;4
15	pDONR221-L1-bCA4.1-C109A-WO-L4	1;4
16	pDONR221-L1-bCA4.1-D111A-WO-L4	1;4
17	pDONR221-L1-bCA4.1-R113A-WO-L4	1;4
18	pDONR221-L1-bCA4.1-I133A-WO-L4	1;4
19	pDONR207-L1-bCA4.1-N36A-ST-L2	11;12
20	pDONR207-L1-bCA4.1-T41A-ST-L2	13;14
21	pDONR207-L1-bCA4.1-K61A-ST-L2	15;16
22	pDONR207-L1-bCA4.1-N67A-ST-L2	17;18
23	pDONR207-L1-bCA4.1-T76A-ST-L2	19;20
24	pDONR207-L1-bCA4.1-T79A-ST-L2	21;22
25	pDONR207-L1-bCA4.1-Q80A-ST-L2	23;24
26	pDONR207-L1-bCA4.1-S90A-ST-L2	25;26
27	pDONR207-L1-bCA4.1-H95A-ST-L2	27;28
28	pDONR207-L1-bCA4.1-T99A-ST-L2	29;30
29	pDONR207-L1-bCA4.1-Q100A-ST-L2	31;32
30	pDONR207-L1-bCA4.1-T101A-ST-L2	33;34
31	pDONR207-L1-bCA4.1-P102A-ST-L2	35;36
32	pDONR207-L1-bCA4.1-K103A-ST-L2	37;38
33	pDONR207-L1-bCA4.1-C109A-ST-L2	39;40
34	pDONR207-L1-bCA4.1-D111A-ST-L2	41;42
35	pDONR207-L1-bCA4.1-R113A-ST-L2	43;44
36	pDONR207-L1-bCA4.1-C115A-ST-L2	45;46
37	pDONR207-L1-bCA4.1-P124A-ST-L2	47;48
38	pDONR207-L1-bCA4.1-F128A-ST-L2	49;50
39	pDONR207-L1-bCA4.1-R131A-ST-L2	51;52
40	pDONR207-L1-bCA4.1-I133A-ST-L2	53;54
41	pDONR207-L1-bCA4.1-Q142A-ST-L2	55;56
42	pDONR207-L1-bCA4.1-K143A-ST-L2	57;58
43	pDONR207-L1-bCA4.1-G147A-ST-L2	59;60
44	pDONR207-L1-bCA4.1-Y154A-ST-L2	61;62
45	pDONR207-L1-bCA4.1-V156A-ST-L2	63;64
46	pDONR207-L1-bCA4.1-V157A-ST-L2	65;66

47	pDONR207-L1-bCA4.1-H158A-ST-L2	67;68
48	pDONR207-L1-bCA4.1-L159A-ST-L2	69;70
49	pDONR207-L1-bCA4.1-K160A-ST-L2	71;72
50	pDONR207-L1-bCA4.1-V161A-ST-L2	73;74
51	pDONR207-L1-bCA4.1-E162A-ST-L2	75;76
52	pDONR207-L1-bCA4.1-N163A-ST-L2	77;78
53	pDONR207-L1-bCA4.1-I164A-ST-L2	79;80
54	pDONR207-L1-bCA4.1-L165A-ST-L2	81;82
55	pDONR207-L1-bCA4.1-V166A-ST-L2	83;84
56	pDONR207-L1-bCA4.1-I167A-ST-L2	85;86
57	pDONR207-L1-bCA4.1-G168A-ST-L2	87;88
58	pDONR207-L1-bCA4.1-H169A-ST-L2	89;90
59	pDONR207-L1-bCA4.1-S170A-ST-L2	91;92
60	pDONR207-L1-bCA4.1-C172A-ST-L2	93;94
61	pDONR207-L1-bCA4.1-G173A-ST-L2	95;96
62	pDONR207-L1-bCA4.1-Q189A-ST-L2	97;98
63	pDONR207-L1-bCA4.1-S190A-ST-L2	99;100
64	pDONR207-L1-bCA4.1-E209A-ST-L2	101;102
65	pDONR207-L1-bCA4.1-K222A-ST-L2	103;104
66	pDONR207-L1-bCA4.1-V245A-ST-L2	105;106
67	pDONR207-L1-bCA4.1-V246A-ST-L2	107;108
68	pDONR207-L1-bCA4.1-K247A-ST-L2	109;110
69	pDONR207-L1-bCA4.1-N248A-ST-L2	111;112
70	pDONR207-L1-bCA4.1-T249A-ST-L2	113;114
71	pDONR207-L1-bCA4.1-L250A-ST-L2	115;116
72	pDONR207-L1-bCA4.1-G255A-ST-L2	117;118
73	pDONR207-L1-bCA4.1-Y257A-ST-L2	119;120
74	pDONR207-L1-bCA4.1-F279A-ST-L2	121;122
75	pDONR207-L1-bCA4.1-T79A_Q80A-ST-L2	123;124
76	pDONR207-L1-bCA4.1-Q142A_K143A-ST-L2	125;126
77	pDONR207-L1-bCA4.1-Q189A_S190A-ST-L2	127;128
78	pDONR207-L1-bCA4.1-V157A_H158A-ST-L2	129;130
79	pDONR207-L1-bCA4.1-V157A_K160A-ST-L2	131;132
80	pDONR207-L1-bCA4.1-H158A_K160A-ST-L2	133;134
81	pDONR207-L1-bCA4.1-V157A_H158A_K160A-ST-L2	135;136
82	pDONR207-L1-bCA4.1-T83A_E84A_K85A_Y86A-ST-L2	137;138
83	pDONR207-L1-bCA4.1-K247A_N248A_T249A_L250A-S	139;140
84	pDONR207-L1-bCA4.1-V161A_E162A-ST-L2	141;142
85	pDONR207-L1-bCA4.1-Q100A_T101A_P102A_K103A-S	143;144
86	pDONR207-L1-bCA4.1-E84A_K85A-ST-L2	145;146
87	pDONR207-L1-bCA4.1-H169A_C172A-ST-L2	147;148
88	pDONR207-L1-bCA4.1-D111A_R113A-ST-L2	149;150
89	pENTR5-L4-bCA4.1pro-R1	151;152
90	pDONR221-R2-myc-ST-L3	153;154

Note: -ST: with stop codon; -WO: without stop codon

Supplementary Data 1. Primers and entry clones - Primers

Entry clones

1	GW-S-B1-bCA4.1	ggggacaagtttgtacaaaaaagcaggcttaATGGCTCCTGCATTCGGAAA
2	GW-S-B1-bCA4.2	ggggacaagtttgtacaaaaaagcaggcttaATGGCAACGGAATCGTACGAA
3	GW-A-B2-bCA4-ST	ggggaccactttgtacaagaaagctgggtTTAAGAGAAGGCAAAAGCA
4	GW-A-B4-bCA4-WO	ggggacaactttgtatagaaaagttgggtaAGAGAAGGCAAAAGCAGGA
5	GW-S-B1-bCA1	ggggacaagtttgtacaaaaaagcaggcttaATGTCGACCGCTCCTCTCT
6	GW-A-B2-bCA1-ST	ggggaccactttgtacaagaaagctgggtTTACAGCTTCCAATGTAGTATGGTAGC
7	GW-S-B1-bCA3	ggggacaagtttgtacaaaaaagcaggcttaATGTCGACAGAGTCGTACGAA
8	GW-A-B2-bCA3-ST	ggggaccactttgtacaagaaagctgggtTTAAGACAAGGCAAAGGCAGG
9	GW-A-B4-bCA3-WO	ggggacaactttgtatagaaaagttgggtaAGACAAGGCAAAGGCAGGGGT
10	GW-S-B1-SLAC1	ggggacaagtttgtacaaaaaagcaggcttaATGGAGAGGAAACAGTCAAATGCT
11	GW-A-B2-SLAC1-WO	ggggaccactttgtacaagaaagctgggtGTGATGCGACTCTTCCTCTGC
12	GW-S-B3-SLAC1	ggggacaactttgtataataaagttgtaATGGAGAGGAAACAGTCAAATGCT

Note: -ST: with stop codon; -WO: without stop codon

SDM clones

11	SDM-S-bCA4.1-N36A	CCATTAAAGGCCTCGCTGATCTTCTCAGTAC
12	SDM-A-bCA4.1-N36A	GAAGATCAGCGAGGCCTTTAATGGCG
13	SDM-S-bCA4.1-T41A	AATGATCTCTTAAGTGCGAAAGCGGATCTCG
14	SDM-A-bCA4.1-T41A	CCGCTTTCGCACTTAAGAGATCATTGAGTCC
15	SDM-S-bCA4.1-K61A	CGGAGCTAGCGGAGCTTGACTCAAG
16	SDM-A-bCA4.1-K61A	CAAGCTCCGCTAGCTCCGCCGTC
17	SDM-S-bCA4.1-N67A	GACTCAAGCGCTTCAGACGCAATTGAAC
18	SDM-A-bCA4.1-N67A	GCGTCTGAAGCGCTTGAGTCAAGCTC
19	SDM-S-bCA4.1-T76A	ACGAATCAAGGCCGGTTTTACTCA
20	SDM-A-bCA4.1-T76A	AAAACCGGCCTTGATTCGTTCAATTGC
21	SDM-S-bCA4.1-T79A	CCGGTTTTGCGCAATTCAAAACCG
22	SDM-A-bCA4.1-T79A	TTGAATTGCGCAAAACCGGTCTTG
23	SDM-S-bCA4.1-Q80A	CGGTTTTACTGCATTTAAAACCGAGAAATATT
24	SDM-A-bCA4.1-Q80A	CTCGGTTTTAAATGCAGTAAAACCGGTC
25	SDM-S-bCA4.1-S90A	CGAGAAATATCTTAAGAATGCTACTTTGTTCAATCATC
26	SDM-A-bCA4.1-S90A	AACAAAGTAGCATTCTTAAGATATTTCTCGGTT
27	SDM-S-bCA4.1-H95A	TTGTTCAATGCTCTTGCCAAGACTCA
28	SDM-A-bCA4.1-H95A	TGGCAAGAGCATTGAACAAAGTAC
29	SDM-S-bCA4.1-T99A	TTGCCAAGGCTCAGACCCCAA
30	SDM-A-bCA4.1-T99A	GGTCTGAGCCTTGGCAAGATG
31	SDM-S-bCA4.1-Q100A	CCAAGACTGCGACCCCAAAGTTTCTGG
32	SDM-A-bCA4.1-Q100A	TTGGGGTCGCAGTCTTGGCAAGATG
33	SDM-S-bCA4.1-T101A	CAAGACTCAGGCCCCAAAGTTTCTGG

34	SDM-A-bCA4.1-T101A	CTTTGGGGCCTGAGTCTTGGCA
35	SDM-S-bCA4.1-P102A	GACTCAGACCGCAAAGTTTCTGGTG
36	SDM-A-bCA4.1-P102A	AGAAACTTTGCGGTCTGAGTCTTGGC
37	SDM-S-bCA4.1-K103A	AGACCCCAGCGTTTCTGGTGTTTGCT
38	SDM-A-bCA4.1-K103A	CCAGAAACGCTGGGGTCTGAGTCT
39	SDM-S-bCA4.1-C109A	GGTGTTTGCTGCCTCTGATTCTCGAG
40	SDM-A-bCA4.1-C109A	AATCAGAGGCAGCAAACACCAG
41	SDM-S-bCA4.1-D111A	CTTGCTCTGCTTCTCGCGTTTGTCCATCTC
42	SDM-A-bCA4.1-D111A	ATGGACAAACGCGAGAAGCAGAGCAAGCAAACACC
43	SDM-S-bCA4.1-R113A	TCTGATTCTGCAGTTTGTCCATCTCACA
44	SDM-A-bCA4.1-R113A	TGGACAAACTGCAGAATCAGAGCAAGC
45	SDM-S-bCA4.1-C115A	CTCTGATTCTAGAGTTGCTCCATCTCACATC
46	SDM-A-bCA4.1-C115A	GTGAGATGGAGCAACTCTAGAATCAGAGCAA
47	SDM-S-bCA4.1-P124A	GAATTTCCAAGCTGGTGAGGCTTTTGTTG
48	SDM-A-bCA4.1-P124A	GCCTCACCAGCTTGGAAATTCAAGATG
49	SDM-S-bCA4.1-F128A	GGTGAGGCTGCAGTTGTCAGAAACATAGCC
50	SDM-A-bCA4.1-F128A	TTCTGACAACTGCAGCCTCACCAGG
51	SDM-S-CA4.1-R131A	TTGTTGTCGCGAACATAGCCAATATGG
52	SDM-A-CA4.1-R131A	TGGCTATGTTCGCGACAACAAAAGCCT
53	SDM-S-bCA4.1-I133A	TGTCAGAAACGCAGCCAATATGGTTCCAC
54	SDM-A-bCA4.1-I133A	TATTGGCTGCGTTTCTGACAAC
55	SDM-S-bCA4.1-Q142A	CCTTTTGACGCGAAGAGACACTCTGG
56	SDM-A-bCA4.1-Q142A	GTGTCTCTTCGCGTCAAAAGGTGG
57	SDM-S-bCA4.1-K143A	ACCTTTTGATCAGGCGAGACACTCTGGAG
58	SDM-A-bCA4.1-K143A	AGTGTCTCGCCTGATCAAAAGGTGGAA
59	SDM-S-bCA4.1-G147A	GACACTCTGCAGTTGGCGCCGC
60	SDM-A-bCA4.1-G147A	GCGCCAACTGCAGAGTGTCTCTTCTGG
61	SDM-S-bCA4.1-Y154A	CGCCGTTGAAGCTGCAGTTGTACATCTC
62	SDM-A-bCA4.1-Y154A	TACAACTGCAGCTTCAACGGCGGC
63	SDM-S-bCA4.1-V156A	GAATACGCAGCTGTACATCTCAAGGTGG
64	SDM-A-bCA4.1-V156A	GAGATGTACAGCTGCGTATTCAACGG
65	SDM-S-bCA4.1-V157A	CGCAGTTGCACATCTTAAGGTGGAGAAC
66	SDM-A-bCA4.1-V157A	CTCCACCTTAAGATGTGCAACTGCGTATTCAAC
67	SDM-S-bCA4.1-H158A	CGCAGTTGTAGCGCTCAAGGTGGAG
68	SDM-A-bCA4.1-H158A	ACCTTGAGCGCTACAACTGCGTATTCAAC
69	SDM-S-bCA4.1-L159A	GTTGTACATGCCAAGGTGGAGAACATT
70	SDM-A-bCA4.1-L159A	CCACCTTGGCATGTACAACTGCGT
71	SDM-S-bCA4.1-K160A	ACGCAGTTGTGCACCTCGCGGTGGAGAACATT
72	SDM-A-bCA4.1-K160A	TCTCCACCGCGAGGTGCACAACTGCGTATTCAAC
73	SDM-S-bCA4.1-V161A	ATCTCAAGGCGGAGAATATTTTGGTGATAGGC

74	SDM-A-bCA4.1-V161A	TCACCAAAATATTCTCCGCCTTGAGATGTAC
75	SDM-S-bCA4.1-E162A	CTCAAGGTGGCCAACATTTTGGTG
76	SDM-A-bCA4.1-E162A	AAAATGTTGGCCACCTTGAGATGTACAA
77	SDM-S-bCA4.1-N163A	AAGGTGGAGGCCATTTTGGTGATAGGC
78	SDM-A-bCA4.1-N163A	CCAAAATGGCCTCCACCTTGAGATGTAC
79	SDM-S-bCA4.1-I164A	GGTGGAGAACGCTTTGGTGATAGGC
80	SDM-A-bCA4.1-I164A	TCACCAAAGCGTTCTCCACCTTGAGATG
81	SDM-S-bCA4.1-L165A	GAGAACATTGCGGTGATAGGCCATAGCTG
82	SDM-A-bCA4.1-L165A	GCCTATCACCGCAATGTTCTCCAC
83	SDM-S-bCA4.1-V166A	CATTTTGGCCATAGGCCATAGCTG
84	SDM-A-bCA4.1-V166A	TGGCCTATGGCCAAAATGTTCTCC
85	SDM-S-bCA4.1-I167A	ATTTTGGTGGCCGGCCATAGCTGC
86	SDM-A-bCA4.1-I167A	AGCTATGGCCGGCCACCAAAATGTTC
87	SDM-S-bCA4.1-G168A	GGTGATAGCCCACAGCTGCTGTGGT
88	SDM-A-bCA4.1-G168A	CAGCAGCTGTGGGCTATCACCAAAATGTT
89	SDM-S-bCA4.1-H169A	GTGATAGGCGCTAGCTGCTGTGGTG
90	SDM-A-bCA4.1-H169A	CAGCAGCTAGCGCCTATCACCAA
91	SDM-S-bCA4.1-S170A	TAGGCCATGCCTGCTGTGGTGGTA
92	SDM-A-bCA4.1-S170A	ACCACAGCAGGCATGGCCTATCACC
93	SDM-S-bCA4.1-C172A	CCATAGCTGCGCAGGTGGTATTAAGG
94	SDM-A-bCA4.1-C172A	ATACCACCTGCGCAGCTATGGCCT
95	SDM-S-bCA4.1-G173A	GCTGCTGTGCTGGTATTAAGGGACTC
96	SDM-A-bCA4.1-G173A	TTAATACCAGCACAGCAGCTATGGCCT
97	SDM-S-bCA4.1-Q189A	GCCCCAACTGCAAGTGACTTCATTGAA
98	SDM-A-bCA4.1-Q189A	GAAGTCACTTGCAGTTGGGGCAGC
99	SDM-S-bCA4.1-S190A	CCAACTCAAGCTGACTTCATTGA
100	SDM-A-bCA4.1-S190A	AATGAAGTCAGCTTGAGTTGGGGCAG
101	SDM-S-bCA4.1-E209A	AAGATCAAGGCGGAACATAAAGACTTGAG
102	SDM-A-bCA4.1-E209A	TTATGTTCCGCCTTGATCTTGTTCCTC
103	SDM-S-bCA4.1-K222A	AATGCAACGCGTGTGAGAAGGAAGCTG
104	SDM-A-bCA4.1-K222A	CTTCTCACACGCGTTGCATTGATCATC
105	SDM-S-bCA4.1-V245A	AGAGCTGAGGCGGTGAAGAACACA
106	SDM-A-bCA4.1-V245A	TTCTTCACCGCCTCAGCTCTCACG
107	SDM-S-bCA4.1-V246A	GCTGAGGTGGCGAAGAACACACTTG
108	SDM-A-bCA4.1-V246A	TGTTCTTCGCCACCTCAGCTCTCACG
109	SDM-S-bCA4.1-K247A	TGAGGTGGTGGCGAACACACTTGC
110	SDM-A-bCA4.1-K247A	AAGTGTGTTCGCCACCACCTCAGCTC
111	SDM-S-bCA4.1-N248A	TGGTGAAGGCCACACTTGCAATAAGAGG
112	SDM-A-bCA4.1-N248A	TGCAAGTGTGGCCTTCACCACCTC
113	SDM-S-bCA4.1-T249A	GGTGAAGAACGCACTTGCAATAAGAGG

114	SDM-A-bCA4.1-T249A	TTGCAAGTGCGTTCTTCACCACCTC
115	SDM-S-bCA4.1-L250A	AAGAACACAGCTGCAATAAGAGGAGGTC
116	SDM-A-bCA4.1-L250A	TTATTGCAGCTGTGTTCTTCACCACCTC
117	SDM-S-bCA4.1-G255A	TAAGAGGAGCTCACTACAATTTCGTC
118	SDM-A-bCA4.1-G255A	TTGTAGTGAGCTCCTCTTATTGCAAGTG
119	SDM-S-bCA4.1-Y257A	GGAGGTCACGCCAATTTCGTCAAAGG
120	SDM-A-bCA4.1-Y257A	CGAAATTGGCGTGACCTCCTCTTATTGC
121	SDM-S-bCA4.1-F279A	TGCTTTTGCCGCCTCATGAACCCAGCTTTCTTGTAC
122	SDM-A-bCA4.1-F279A	GCTGGGTTCATGAGGCGGCAAAAGCAGGAG
123	SDM-S-bCA4.1-T79A_Q80A	CCGGTTTTGCTGCATTTAAAACCGAGAAA
124	SDM-A-bCA4.1-T79A_Q80A	TCGGTTTTAAATGCAGCAAAACCGGTCTT
125	SDM-S-bCA4.1-Q142A_K143A	CTTTTGACGCGGCGAGACACTCTGG
126	SDM-A-bCA4.1-Q142A_K143A	AGAGTGTCTCGCCGCGTCAAAAGGTGG
127	SDM-S-bCA4.1-Q189A_S190A	GCCCCAACTGCAGCTGACTTCATTGA
128	SDM-A-bCA4.1-Q189A_S190A	ATGAAGTCAGCTGCAGTTGGGGCAGC
129	SDM-S-bCA4.1-V157A_H158A	ACGCAGTTGCAGCGCTCAAGGTGGAGAAC
130	SDM-A-bCA4.1-V157A_H158A	CCACCTTGAGCGCTGCAACTGCGTATTCAACGG
131	SDM-S-bCA4.1-V157A_K160A	CGCAGTTGCGCATCTCGCGGTGGAGAACATT
132	SDM-A-bCA4.1-V157A_K160A	TCTCCACCGCGAGATGCGCAACTGCGTATTCAACGG
133	SDM-S-bCA4.1-H158A_K160A	CGCAGTTGTAGCGCTCGCGGTGGAGAACATT
134	SDM-A-bCA4.1-H158A_K160A	TCTCCACCGCGAGCGCTACAACTGCGTATTCAAC
135	SDM-S-bCA4.1-V157A_H158A_K160A	ACGCAGTTGCAGCGCTCGCGGTGGAGAACATT
136	SDM-A-bCA4.1-V157A_H158A_K160A	TCTCCACCGCGAGCGCTGCAACTGCGTATTCAAC
137	SDM-S-bCA4.1-T83A_E84A_K85A_Y86A	TCAATTCAAAGCCGCGGCAGCTTTGAAGAATAGTAC
138	SDM-A-bCA4.1-T83A_E84A_K85A_Y86A	TTCTTCAAAGCTGCCGCGGCTTTGAATTGAGT
139	SDM-S-bCA4.1-K247A_N248A_T249A_L250A	TGAGGTGGTGGCGGCCGCAGCTGCAATAAGAGGAGGTC
140	SDM-A-bCA4.1-K247A_N248A_T249A_L250A	TTATTGCAGCTGCGGCCGCCACCACCTCAGCTC
141	SDM-S-bCA4.1-V161A_E162A	GTACATCTTAAGGCGGCGAACATTTTGGTG
142	SDM-A-bCA4.1-V161A_E162A	AAATGTTCGCCGCCTTAAGATGTACAACTGC
143	SDM-S-bCA4.1-Q100A_T101A_P102A_K103A	GCCAAGACTGCGGCCGCGGCGTTTCTGGTGTTTGCTT
144	SDM-A-bCA4.1-Q100A_T101A_P102A_K103A	ACCAGAAACGCCGCGGCCGCAGTCTTGGCAAGATGAT
145	SDM-S-bCA4.1-E84A_K85A	CAAAACCGCGGCATATTTGAAGAATAGTAC
146	SDM-A-bCA4.1-E84A_K85A	CTTCAAATATGCCGCGGTTTTGAATTGAGT
147	SDM-S-bCA4.1-H169A_C172A	GGTGATAGGCGCTAGCTGCGCTGGTGGTATTAAGG
148	SDM-A-bCA4.1-H169A_C172A	TACCACCAGCGCAGCTAGCGCCTATCACCAA
149	SDM-S-bCA4.1-D111A_R113A	TTGCTCTGCTTCTGCAGTTTGTCCATCTCA
150	SDM-A-bCA4.1-D111A_R113A	GGACAAACTGCAGAAGCAGAGCAAGCAA

Note: Green letters restriction enzyme site; Red letters mutagenesis

multisite cloning

151	SacI-bCA4.1pro-F	cttgagctcTTCGCAAGTGTAAACGCAGA
152	MfeI-bCA4.1pro-R	taacaattgTATAAATGATCTCTTCCTTTGCTTC
153	MGW-S-B2r-myc	ggggacagctttcttgtacaaagtggtaATGAAGCTGATCTCAGAGGAGGACCTGTGA
154	MGW-A-B3-myc-st	ggggacaactttgtataataaagttggTCACAGGTCCTCCTCTGAGATCAGCTTCAT
