# Supplementary material for: A guard cell carbonic anhydrase binds and regulates SLAC1 separate from its catalytic activity
Source: Nat Commun. 2026 Mar 13;17:3911. doi: 10.1038/s41467-026-70596-9 (PMC13128974; doi:10.1038/s41467-026-70596-9)
Supplement: Supplementary file 4 — Supplementary Data 2 [file 41467_2026_70596_MOESM4_ESM.pdf]

Supplementary Data 2. OnGuard3 model parameters
Parameters in **bold** are for the mutant with reduced sensitivity to CO_2/HCO_3^-.

Total cell volume = 0.335605 pL; vacuolar fraction = 83.6828%

Stomatal Aperture Parameters:
SA:P 'm'= 0.8 atm/µm; SA:P 'n'= 3 atm; SA:V 'R'= 0.05 pL/µm SA:V 'S'= 0.3 pL
Pore Length: 8 µm; Pore Depth: 15 µm

Cytosolic Protein Buffering: [Pr] = 0.119228 mM, pIso = 6.8, ap = -71
Cytosolic Calcium Buffering: [Bu] = 0.263594 mM, K = 3e-06 M # Ca Sites = 10

Temperature: Leaf: 25∞C; Air: 25∞C
Current 'time' in model = 00:00:00.00

*** Compartmental Solutions (/mM)
==================================

	Apoplast	Cytosol	Vacuole
pH	6.500000	7.657059	5.293206
K	10.00000	89.35475	16.70185
Ca	1.000000	0.009441	35.86319
Cl	12.00000	15.77964	84.42793
Suc	0.010000	0.013379	0.002872
MH2	3.23e-07	6.18e-08	0.098169
MH	7.94e-06	0.000022	0.149686
M	0.009992	0.393482	11.70544
HCO3	0.023907	0.343228	0.001485
CO2	0.016989	0.016989	0.016989
Malates	0.010000	0.393504	11.95330

*** Plasma Membrane Transporters
=================================

PM K-in Channel [3000 units] (Inward-Rectifying GHK Channel)

#'K' G/Gmax = 9 pOhms
2-State Voltage Gate: VΩ = -185 mV,
Zg = +1.8
Light-Sensitive: NO!
Ligand-Gates:
Ca-inhibited (cytosol): Kd= 3.3e-07, Hill= 4;
H-activated (cytosol): Kd= 6e-08, Hill= 2;
H-activated (apoplast): Kd= 1e-07, Hill= 1;

PM K-out Channel [240 units] (Outward-Rectifying GHK Channel)

#'K' G/Gmax = 20 pOhms
VΩ = +1 ◊ F/RT ◊ ln([K]apo/10mM)
Zg = +2
Light-Sensitive: NO!
Ligand-Gates:
H-inhibited (cytosol): Kd= 3e-08, Hill= 2;
HCO3-activated (cytosol): Kd= 0.0003, Hill= 2;

```
R-Type Anion Channel [50 units] (Outward-Rectifying GHK Channel)
--------------------
#'Cl' G/Gmax = 3.4 pOhms
#'M'  G/Gmax = 2 pOhms
      VΩ = +1 ◊ F/RT ◊ ln(1e-06mM/[H]cyt)
      Zg = -2
Light-Sensitive: NO!
Ligand-Gates:
  Ca-activated (cytosol): Kd= 6e-07, Hill= 4;
  H-activated (cytosol): Kd= 3e-08, Hill= 3;
  HCO3-activated (cytosol): Kd= 0.0003, Hill= 3;


R-Type Anion Channel voltage-independent component [100 units]
 ---------
#'Cl' G/Gmax = 0.15 pOhms
#'M'  G/Gmax = 0.07 pOhms
      Voltage-Independent
Light-Sensitive: NO!
Ligand-Gates:
  Ca-activated (cytosol): Kd= 6e-07, Hill= 4;
  H-activated (cytosol): Kd= 4e-08, Hill= 3;
  HCO3-activated (cytosol): Kd= 0.0003, Hill= 3;



SLAC1 Anion Channel [900 units] (Outward-Rectifying GHK Channel)
--------
#'Cl' G/Gmax = 3.4 pOhms
#'M'  G/Gmax = 2 pOhms
      VΩ = +1 ◊ F/RT ◊ ln(1e-06mM/[H]cyt)
      Zg = -2
Light-Sensitive: NO!
Ligand-Gates:
  Ca-activated (cytosol): Kd= 6e-07, Hill= 4;
  H-activated (cytosol): Kd= 3e-08, Hill= 3;
  HCO3-activated (cytosol): Kd= 0.0003 [=0.003], Hill= 1;



V-Gated Ca-IN [12 units] (Inward-Rectifying GHK Channel)
-------------
#'Ca' G/Gmax = 12 pOhms
      VΩ = +0.5 ◊ F/RT ◊ ln(2e-09mM/[Ca]cyt)
      Zg = +1
Light-Sensitive: NO!
Ligand-Gates:
  Ca-inhibited (cytosol): Kd= 5e-07, Hill= 5;


H-ATPase [300000 units] (4-State 'Slayman' Pump)
--------
#'H'  Stoichiometry = +1; binds at 4->1 (in) and 3->2 (ex);
      K12 = 2000, K23 = 50000,     K34 = 500,  K41 = 2e+09,
      K21 = 100,  K32 = 1e+08,     K43 = 10,   K14 = 200;
Light-Sensitive: Yes: LΩ = 50 µEinsteins, Fmin = 5%
```

```
Ligand-Gates:
  Ca-inhibited (cytosol): Kd= 2.5e-07, Hill= 3;


H:Cl Symport [50000 units] (4-State 'Slayman' Pump)
------------
#'H'  Stoichiometry = +2; binds at 4->1 (in) and 3->2 (ex);
#'Cl' Stoichiometry = +1; binds at 4->1 (in) and 4->3 (ex);
      K12 = 1000, K23 = 100,  K34 = 50000,     K41 = 1e+21,
      K21 = 50,   K32 = 1e+21,     K43 = 100000,    K14 = 100;
Light-Sensitive: NO!
Ligand-Gates:
 <none>


H:K Symport [16000 units] (4-State 'Slayman' Pump)
-----------
#'H'  Stoichiometry = +1; binds at 4->1 (in) and 3->2 (ex);
#'K'  Stoichiometry = +1; binds at 4->1 (in) and 4->3 (ex);
      K12 = 2,    K23 = 10000,     K34 = 100000,    K41 = 1e+14,
      K21 = 0.4,  K32 = 1e+12,     K43 = 1e+10,     K14 = 50;
Light-Sensitive: NO!
Ligand-Gates:
 <none>


Ca-ATPase [60000 units] (4-State 'Slayman' Pump)
---------
#'Ca' Stoichiometry = +1; binds at 4->1 (in) and 3->2 (ex);
      K12 = 2000, K23 = 10000,     K34 = 500,  K41 = 1e+15,
      K21 = 2,    K32 = 1e+07,     K43 = 500,  K14 = 1000;
Light-Sensitive: Yes: LΩ = 50 µEinsteins, Fmin = 50%
Ligand-Gates:
  Ca-activated (cytosol): Kd= 5e-07, Hill= 2;
  HCO3-inhibited (cytosol): Kd= 0.0003, Hill= 4;


HMal symp [40000 units] (Concentration-Driven SYMPORT)
---------
#'H'  (Stoichiometry = -3)
#'M'  (Stoichiometry = -1)
      Fmax = 1e+20
Light-Sensitive: NO!
Ligand-Gates:
 <none>


K leak [1 units] (Inward-Rectifying GHK Channel)
------
#'K'  G/Gmax = 1 pOhms
      Voltage-Independent
Light-Sensitive: NO!
Ligand-Gates:
 <none>



*** Tonoplast Transporters
```

```
==========================

TPK1 [300 units] (Inward-Rectifying Ohmic Channel)
----
#'K'  G/Gmax = 90 pOhms
      Voltage-Independent
Light-Sensitive: NO!
Ligand-Gates:
  Ca-activated (cytosol): Kd= 3e-06, Hill= 1;
  H-activated (cytosol): Kd= 3e-08, Hill= 3;


TPC1 [100 units] (Outward-Rectifying GHK Channel)
----
#'Ca' G/Gmax = 27 pOhms
#'K'  G/Gmax = 14 pOhms
      VΩ = +1 ◊ F/RT ◊ ln([Ca]vac/2mM)
      Zg = +2
Light-Sensitive: NO!
Ligand-Gates:
  Ca-activated (cytosol): Kd= 3e-05, Hill= 1;
  H-activated (vacuole): Kd= 1e-06, Hill= 1;


FV K Channel [1600 units] (Inward-Rectifying GHK Channel)
------------
#'K'  G/Gmax = 6 pOhms
      2-State Voltage Gate: VΩ = -30 mV,
      Zg = +1
Light-Sensitive: NO!
Ligand-Gates:
  Ca-inhibited (cytosol): Kd= 2e-07, Hill= 1;
  H-inhibited (cytosol): Kd= 4e-07, Hill= 1;


VCL [300 units] (Inward-Rectifying GHK Channel)
---
#'Cl' G/Gmax = 40 pOhms
#'M'  G/Gmax = 10 pOhms
      VΩ = +1 ◊ F/RT ◊ ln([H]vac/0.005mM)
      Zg = -1
Light-Sensitive: NO!
Ligand-Gates:
  Ca-activated (cytosol): Kd= 1e-06, Hill= 1;


Vacuole H-ATPase [400000 units] (4-State 'Slayman' Pump)
----------------
#'H'  Stoichiometry = +2; binds at 4->1 (in) and 3->2 (ex);
      K12 = 100,  K23 = 1000, K34 = 0.5,  K41 = 1e+18,
      K21 = 10,   K32 = 1e+08,      K43 = 5,    K14 = 10000;
Light-Sensitive: Yes: LΩ = 50 µEinsteins, Fmin = 10%
Ligand-Gates:
 <none>


Vacuole H-PPase [1200000 units] (4-State 'Slayman' Pump)
---------------
```

```
#'H'  Stoichiometry = +1; binds at 4->1 (in) and 3->2 (ex);
      K12 = 1000, K23 = 1000, K34 = 1e+11,     K41 = 3e+09,
      K21 = 100,  K32 = 5e+09,     K43 = 1e+07,     K14 = 10000;
Light-Sensitive: Yes: LΩ = 50 µEinsteins, Fmin = 10%
Ligand-Gates:
  Ca-inhibited (cytosol): Kd= 1e-07, Hill= 1;
  K-activated (cytosol): Kd= 0.05, Hill= 1;


Slaymanesque Ca Pump [800000 units] (4-State 'Slayman' Pump)
--------------------
#'Ca' Stoichiometry = +1; binds at 4->1 (in) and 3->2 (ex);
      K12 = 3000, K23 = 1000, K34 = 1000, K41 = 1e+09,
      K21 = 0.3,  K32 = 10000,     K43 = 10,   K14 = 10000;
Light-Sensitive: Yes: LΩ = 50 µEinsteins, Fmin = 50%
Ligand-Gates:
  Ca-activated (cytosol): Kd= 3.5e-07, Hill= 3;
  HCO3-inhibited (cytosol): Kd= 0.0003, Hill= 4;
  Ca-inhibited (vacuole): Kd= 0.04, Hill= 4;


Vac.CLC [120000 units] (4-State 'Slayman' Pump)
-------
#'H'  Stoichiometry = +1; binds at 4->1 (in) and 3->2 (ex);
#'Cl' Stoichiometry = -2; binds at 1->4 (in) and 2->3 (ex);
      K12 = 1000, K23 = 1e+09,     K34 = 100,  K41 = 1e+10,
      K21 = 1000, K32 = 1e+09,     K43 = 10,   K14 = 1e+11;
Light-Sensitive: NO!
Ligand-Gates:
  H-inhibited (cytosol): Kd= 5e-08, Hill= 2;


Tonoplast VCa [8 units] (Outward-Rectifying GHK Channel)
-------------
#'Ca' G/Gmax = 10 pOhms
      VΩ = +1 ◊ F/RT ◊ ln(10mM/[Ca]vac)
         + +0.5 ◊ F/RT ◊ ln(0.5mM/[HCO3]cyt)
      Zg = +4
Light-Sensitive: NO!
Ligand-Gates:
  Ca-activated (cytosol): Kd= 5e-07, Hill= 4;
T-deactivation: switch= [Ca]cyt; Threshold= 0.001mM, TΩ= 100000ms, reset=
5%/0.0005mM


CAX [100000 units] (Concentration-Driven ANTIPORT)
---
#'H'  (Stoichiometry = -3)
#'Ca' (Stoichiometry = +1)
      Fmax = 1e+22
Light-Sensitive: NO!
Ligand-Gates:
  Ca-activated (cytosol): Kd= 3e-06, Hill= 1;
  H-inhibited (cytosol): Kd= 4e-08, Hill= 3;


ALMT-Mal [600 units] (Inward-Rectifying GHK Channel)
--------
```

```
#'M'  G/Gmax = 6 pOhms
      2-State Voltage Gate: VΩ = +0 mV,
      Zg = -2
Light-Sensitive: NO!
Ligand-Gates:
  H-inhibited (cytosol): Kd= 8.5e-08, Hill= 2;
  Ca-activated (cytosol): Kd= 1e-06, Hill= 1;


NHX [20000 units] (Concentration-Driven ANTIPORT)
---
#'H'  (Stoichiometry = -1)
#'K'  (Stoichiometry = +1)
      Fmax = 100000
Light-Sensitive: NO!
Ligand-Gates:
 <none>


Vac MLC [0 units] (4-State 'Slayman' Pump)
-------
#'H'  Stoichiometry = +1; binds at 4->1 (in) and 3->2 (ex);
#'M'  Stoichiometry = -1; binds at 1->4 (in) and 2->3 (ex);
      K12 = 50000,     K23 = 2e+07,     K34 = 100,  K41 = 1e+10,
      K21 = 1000, K32 = 1e+09,     K43 = 10,   K14 = 1e+11;
Light-Sensitive: NO!
Ligand-Gates:
  H-inhibited (cytosol): Kd= 5e-08, Hill= 2;


*** METABOLISM
==============

Total Malate  (apo/cyt/vac) = 0.01 0.393504 11.9533 mM
Total Sucrose (apo/cyt/vac) = 0.01 0.0133792 0.00287244 mM

Photosynthesis:
Suc s-max = 10 fmol/h, LΩ = 50 µE
Mal s-max = 0 fmol/h, LΩ = 50 µE
Light Type: Total

Sucrose Sink:
R-max = 10 fmol/h, KΩ = 1 mM

Suc <-> Mal Conversion:
R-max = 5 fmol/h, KΩ(S) = 0.1 mM, KΩ(M) = 10 mM
Mid-point pH = 7.7, pH gradient = +100

'Q10' Temperature Coefficient: 2


*** PHOTOSYNTHESIS & WUE PARAMETERS
===================================

Stomata/mm≤ = 100
Stomatal Length/µm = 8; Depth = 15
Subepidermal Depth/µm: = 300; Empty Space = 50%
```

```
CO2 Assimilation, A = AL ◊ AC - Rd [ Where AL = {fL + Amax-v[(fL +
Amax)≤-4T.fL.Amax]}/2T  and  AC = 1/[1 + Kc/(Cin-Cc)])]

Amax (µmol/m≤/s) = 20
f = 0.15; T = 0.9
Kc (/ppm) = 80; Cc (/ppm) = 8; Rd (µmol/m≤/s) = 2

System used for Ciso = Mott (p-site)
Wet Surface Area Coefficient (RWF) = 40
Mott Potential Attenuator (Divisor) = 24.466


*** Constraint Relaxation & Recovery
====================================

Use CRR? Yes
Solutes to include: K
Epidermal Cell Capacity (Amax, /fmol) = 14
Adjacent Wall Capacity  (Smax, /fmol) = 0.5
Max Recovery Rate (/fmol/sec)  = 0.006; (Order = 1)
Current Contents = 100.0%
Apply Turgor-Sensitivity? Yes
Attenuator Mid-Point (atm) = 7; Attenuator Gradient (/atm) = 2
```
