# Supplementary material for: A guard cell carbonic anhydrase binds and regulates SLAC1 separate from its catalytic activity
Source: Nat Commun. 2026 Mar 13;17:3911. doi: 10.1038/s41467-026-70596-9 (PMC13128974; doi:10.1038/s41467-026-70596-9)
Supplement: Supplementary file 5 — Reporting Summary [file 41467_2026_70596_MOESM5_ESM.pdf]

nature portfolio

Corresponding author(s): Prof. M.R. Blatt

Last updated by author(s): 6. February 2026

Reporting Summary

Nature Portfolio wishes to improve the reproducibility of the work that we publish. This form provides structure for consistency and transparency in reporting. For further information on Nature Portfolio policies, see our Editorial Policies and the Editorial Policy Checklist.

Statistics

For all statistical analyses, confirm that the following items are present in the figure legend, table legend, main text, or Methods section.

n/a	Confirmed	
☐	☒	The exact sample size (*n*) for each experimental group/condition, given as a discrete number and unit of measurement
☐	☒	A statement on whether measurements were taken from distinct samples or whether the same sample was measured repeatedly
☐	☒	The statistical test(s) used AND whether they are one- or two-sided<br>*Only common tests should be described solely by name; describe more complex techniques in the Methods section.*
☒	☐	A description of all covariates tested
☒	☐	A description of any assumptions or corrections, such as tests of normality and adjustment for multiple comparisons
☐	☒	A full description of the statistical parameters including central tendency (e.g. means) or other basic estimates (e.g. regression coefficient) AND variation (e.g. standard deviation) or associated estimates of uncertainty (e.g. confidence intervals)
☐	☒	For null hypothesis testing, the test statistic (e.g. *F*, *t*, *r*) with confidence intervals, effect sizes, degrees of freedom and *P* value noted<br>*Give P values as exact values whenever suitable.*
☒	☐	For Bayesian analysis, information on the choice of priors and Markov chain Monte Carlo settings
☒	☐	For hierarchical and complex designs, identification of the appropriate level for tests and full reporting of outcomes
☒	☐	Estimates of effect sizes (e.g. Cohen's *d*, Pearson's *r*), indicating how they were calculated

Our web collection on statistics for biologists contains articles on many of the points above.

Software and code

Policy information about availability of computer code

Data collection	Data collection utilised existing and published code for imaging, electrophysiology and growth analysis. All software is cited with appropriate references in the text
Data analysis	Data analysis utilised existing and published code for imaging, electrophysiology and growth analysis. All software is cited with appropriate references in the text

For manuscripts utilizing custom algorithms or software that are central to the research but not yet described in published literature, software must be made available to editors and reviewers. We strongly encourage code deposition in a community repository (e.g. GitHub). See the Nature Portfolio guidelines for submitting code & software for further information.

Data

Policy information about availability of data

All manuscripts must include a data availability statement. This statement should provide the following information, where applicable:

- Accession codes, unique identifiers, or web links for publicly available datasets
- A description of any restrictions on data availability
- For clinical datasets or third party data, please ensure that the statement adheres to our policy

Data generated and analysed during this study are included in the article, its supplementary files, and are also available on reasonable request to the corresponding author. The OnGuard3 platform and the model parameter sets are freely available to academic users and may be downloaded from www.plantscienceglasgow.org.

Research involving human participants, their data, or biological material

Policy information about studies with human participants or human data. See also policy information about sex, gender (identity/presentation), and sexual orientation and race, ethnicity and racism.

Reporting on sex and gender	No data or analysis relates to sex or gender
Reporting on race, ethnicity, or other socially relevant groupings	No reporting is included that pertains to race, ethncity or other social groupings
Population characteristics	No human data or related characteristics were included in this study
Recruitment	No participants were recruited
Ethics oversight	No organisation approved this study as it did not include human subjects

Note that full information on the approval of the study protocol must also be provided in the manuscript.

Field-specific reporting

Please select the one below that is the best fit for your research. If you are not sure, read the appropriate sections before making your selection.

[x] Life sciences [ ] Behavioural & social sciences [ ] Ecological, evolutionary & environmental sciences

For a reference copy of the document with all sections, see nature.com/documents/nr-reporting-summary-flat.pdf

Life sciences study design

All studies must disclose on these points even when the disclosure is negative.

Sample size	Sample sizes were chosen to achieve a maximum within the context of experimental and resource feasibility
Data exclusions	No data were excluded
Replication	All experiments were replicated successfully and validated independently through relevant methods as documented in the submission. Electrophysiological data, for example, were validated through analysis of current kinetics and concurrent analysis of related channel currents. Gas exchange data were validated through concurrent measurements of associated experimental parameters, including leaf temperature, IRGA matching, and machine parameters of CO2 and related gas flow variables. Numbers of independent experiments are indicated in the text and the figure legends.
Randomization	Samples were grouped for comparison by genotype and were therefore not randomised at this level. Plants of all types were grown, randomised and were circulated at intervals within the growth facilities to avoid possible positional variables. Plants were prepared and handled in parallel and over the same time of day to ensure identical treatments.
Blinding	Blinding was not possible, because of the visible physiological characteristics associated with the genotypes.

Reporting for specific materials, systems and methods

We require information from authors about some types of materials, experimental systems and methods used in many studies. Here, indicate whether each material, system or method listed is relevant to your study. If you are not sure if a list item applies to your research, read the appropriate section before selecting a response.

Materials & experimental systems

n/a	Involved in the study	
[ ]	[x]	Antibodies
[x]	[ ]	Eukaryotic cell lines
[x]	[ ]	Palaeontology and archaeology
[x]	[ ]	Animals and other organisms
[x]	[ ]	Clinical data
[x]	[ ]	Dual use research of concern
[ ]	[x]	Plants

Methods

n/a	Involved in the study	
[x]	[ ]	ChIP-seq
[x]	[ ]	Flow cytometry
[x]	[ ]	MRI-based neuroimaging

Antibodies

Antibodies used	Antibodies against SLAC1 and the carbonic anhydrases were generated in house and are documented in the supporting information. All other antibodies were obtained from commercial sources as indicated. Antibody dilutions are indicated in the text.
Validation	Please see the cited references. For the SLAC1 and carbonic anhydrase antibodies, refer to Supplemental Figs 2 and 3 for details of validations.

Dual use research of concern

Policy information about dual use research of concern

Hazards

Could the accidental, deliberate or reckless misuse of agents or technologies generated in the work, or the application of information presented in the manuscript, pose a threat to:

No	Yes	
☒	☐	Public health
☒	☐	National security
☒	☐	Crops and/or livestock
☒	☐	Ecosystems
☒	☐	Any other significant area

Experiments of concern

Does the work involve any of these experiments of concern:

No	Yes	
☒	☐	Demonstrate how to render a vaccine ineffective
☒	☐	Confer resistance to therapeutically useful antibiotics or antiviral agents
☒	☐	Enhance the virulence of a pathogen or render a nonpathogen virulent
☒	☐	Increase transmissibility of a pathogen
☒	☐	Alter the host range of a pathogen
☒	☐	Enable evasion of diagnostic/detection modalities
☒	☐	Enable the weaponization of a biological agent or toxin
☒	☐	Any other potentially harmful combination of experiments and agents

Plants

Seed stocks	Tobacco (Nicotiana tabacum) were grown and leaves transformed by Agrobacterium infiltration (Tyrrell et al., 2007). Arabidopsis (Arabidopsis thaliana) wild-type Col0, and the ca1ca4 double mutant (Hu et al., 2010) were from the Nottingham Arabidopsis Stock Centre. Citations are included with appropriate reference in the text.
Novel plant genotypes	Stable transgenic lines were generated in the ca1ca4 mutant by floral dipping and the T1 generation selected following growth on 25 mg/L hygromycin. At least two independent lines per construct were carried forward to the T2 generation and were also used for analysis.
Authentication	Stable plant lines and all yeast constructs were validated by immunochemical analysis. Full descriptions are included in the text and validations presented in Supplemental Fig. 11
